# Supplementary figures and images for: Job satisfaction mediates the effect of self-efficacy on work engagement among physical education teachers in economically disadvantaged areas
Source: PLoS One. 2025 Apr 17;20(4):e0321055. doi: 10.1371/journal.pone.0321055 (PMC12005525; doi:10.1371/journal.pone.0321055)

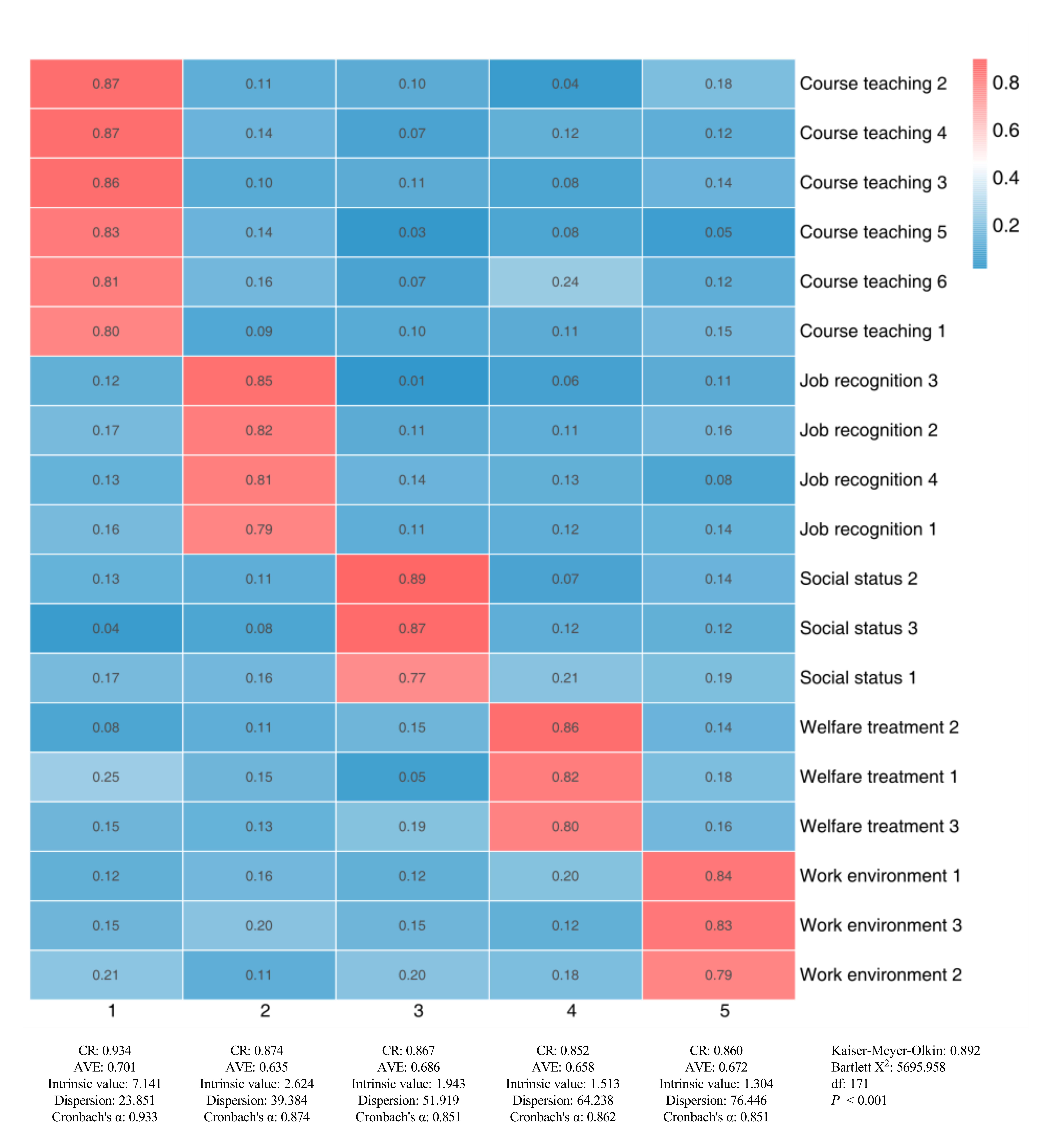

Supplement: S2 Fig — (TIF) [file pone.0321055.s002.tif]

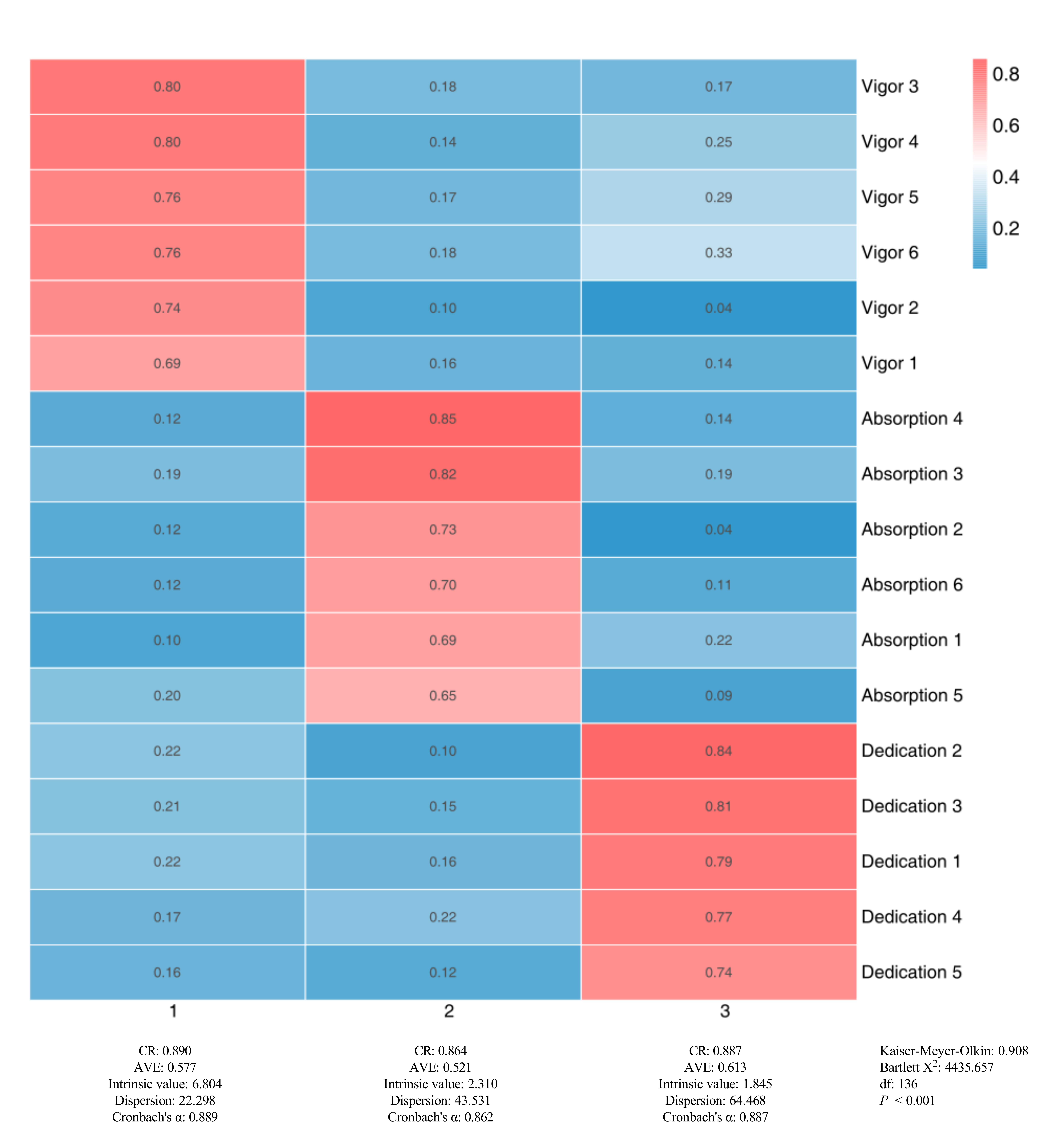

Supplement: S3 Fig — (TIF) [file pone.0321055.s003.tif]
