# Supplementary material for: Job satisfaction mediates the effect of self-efficacy on work engagement among physical education teachers in economically disadvantaged areas
Source: PLoS One. 2025 Apr 17;20(4):e0321055. doi: 10.1371/journal.pone.0321055 (PMC12005525; doi:10.1371/journal.pone.0321055)
Supplement: S1 Table — (DOCX) [file pone.0321055.s004.docx]

| **S1 Table.** Compositions of questionnaire | |
| --- | --- |
| Variables | Questions |
| Sociodemographic variables, questions, and scales used in the questionnaires | |
| Age (years) (1: 20-30 years old; 2: 31-40 years old; 3: 41-50 years old; 4: 51 years and above) | How old are you? |
| Gender (1: male; 2: female) | What is your gender? |
| Educational background (1: college degree; 2: bachelor degree; 3: master degree; 4: doctoral degree) | What is your educational background? |
| Professional title: (1: junior; 2: medium; 3: senior) | What is your professional title? |
| Contract type (1: full-time; 2: part-time) | What is your contract type? |
| Weekly teaching hours (1: less than 10 times; 2: 10-15 times; 3: 16-20 times; 4: 21 times and above) | How many hours of class do you have in a week? (without competition organization, extracurricular sports activities guidance, and extracurricular sports training, etc.) |
| Monthly salary (1: 2000-3000 yuan; 2: 3001-4000 yuan; 3: 4001-5000 yuan; 4: 5001 yuan and above) | How much money do you earn in a week? |
| Variables, questions, and scales of self-efficacy used in the questionnaires (Tschannen-Moran and Hoy, 2001) | |
| Instructional strategies (1-5: very dissatisfied – very satisfied) | 1. To what extent can you use various assessment strategies? 2. To what extent can you provide an alternative explanation or example when students feel confused? 3. To what extent can you encourage your students to use self-regulated learning strategies? 4. How capable are you of implementing alternative strategies in the classroom? 5. To what extent can you inform students about the importance and usefulness of self-directed learning strategies? 6. To what extent can you teach your students how to use and apply different self-regulated learning strategies? 7. To what extent can you measure students' understanding of the content you teach? 8. To what extent can you provide appropriate challenges for highly capable students? |
| Classroom management (1-5: very dissatisfied – very satisfied) | 1. To what extent can you control disruptive behavior in the classroom? 2. What can you do to ensure that children follow classroom rules? 3. What can you do to calm down a disruptive or noisy student? 4. To what extent can you establish a classroom management system for each group of students? 5. To what extent can you avoid disruptive behavior from a few students disrupting the entire class? 6. How can you respond to challenging students? 7. To what extent can you clearly communicate your expectations for student behavior? 8. To what extent can you establish routines to keep activities running smoothly? |
| Student engagement (1-5: very dissatisfied – very satisfied) | 1. What can you do to make students believe they can do their homework well? 2. What can you do to help your students value learning? 3. What can you do to motivate students who are not interested in academics? 4. To what extent can you help families support their children in achieving good grades at school? 5. What can you do to improve understanding for students who are failing? 6. What can you do to help your students think critically? 7. What can you do to foster creativity in students? 8. How much can you do to help the most difficult students to get along with? |
| Variables, questions, and scales of job satisfaction used in the questionnaires (Chen and Sun, 2023; Bian, 2023; Huang, 2024) | |
| Course teaching (1-5: very dissatisfied – very satisfied) | 1. How much do you think teaching well impacts your career development? 2. Are you satisfied with the time and effort you invest in teaching? 3. Are you satisfied with the curriculum system? 4. Are you satisfied with the workload of teaching? 5. Are you satisfied with the preparation time and frequency? 6. Are you satisfied with how students respond in class? |
| Welfare treatment (1-5: very dissatisfied – very satisfied) | 1. Are you satisfied with the salary level of physical education teachers at this school? 2. Are you satisfied with the opportunities for career advancement for physical education teachers at this school? 3. Are you satisfied with the fairness and reasonableness of the system for evaluating professional titles for physical education teachers at this school? |
| Work environment (1-5: very dissatisfied – very satisfied) | 1. Are you satisfied with the campus culture atmosphere? 2. Are you satisfied with the teaching environment at the school? 3. Are you satisfied with the office conditions at the school? |
| Job recognition (1-5: very dissatisfied – very satisfied) | 1. How much importance do you think school leadership places on physical education? 2. Are you satisfied with your relationship with school leadership? 3. How much recognition do you feel your colleagues give you? 4. Are you satisfied with how well you cooperate and communicate with your colleagues? |
| Social status (1-5: very dissatisfied – very satisfied) | 1. How do you perceive the societal evaluation of rural physical education teachers? 2. Are you satisfied with the social recognition of the status of rural physical education teachers? 3. Are you satisfied with the social status of rural physical education teachers? |
| Variables, questions, and scales of work engagement used in the questionnaires (Schaufeli et al., 2002) | |
| Vigor (1-5: very dissatisfied – very satisfied) | 1. I always feel energetic while working. 2. I often feel strong and full of vitality in my work. 3. When I wake up in the morning, I look forward to going to work. 4. I can work for long periods of time without feeling tired. 5. I have strong psychological resilience in my work. 6. Even when things are not going well in my work, I always persevere. |
| Dedication (1-5: very dissatisfied – very satisfied) | 1. I believe that my work is meaningful and purposeful. 2. I am passionate about my work. 3. I feel that my work motivates me. 4. I am proud to be a rural physical education teacher. 5. I find the work of a rural physical education teacher challenging. |
| Absorption (1-5: very dissatisfied – very satisfied) | 1. Time passes quickly when I'm working. 2. I become completely absorbed in my work. 3. I feel happy when I work hard. 4. I am deeply engaged in the work of a rural physical education teacher. 5. I always feel excited when I'm working. 6. It's always hard for me to stop working. |
