# Supplementary material for: Job satisfaction mediates the effect of self-efficacy on work engagement among physical education teachers in economically disadvantaged areas
Source: PLoS One. 2025 Apr 17;20(4):e0321055. doi: 10.1371/journal.pone.0321055 (PMC12005525; doi:10.1371/journal.pone.0321055)
Supplement: Attachment — Submitted filename: PONE-D-24-42818_reviewer_120124.pdf [file pone.0321055.s005.pdf]

PLOS ONE

Job satisfaction mediates effect of self-efficacy on work engagement for teachers in economically disadvantaged areas

--Manuscript Draft--

**Manuscript Number:**	PONE-D-24-42818
**Article Type:**	Research Article
**Full Title:**	Job satisfaction mediates effect of self-efficacy on work engagement for teachers in economically disadvantaged areas
**Short Title:**	work engagement & rural teacher
**Corresponding Author:**	Shi Qi Xu<br>Sangmyung University - Seoul Campus: Sangmyung University<br>Cheonan, KOREA, REPUBLIC OF
**Keywords:**	rural teacher; Work Engagement; Job satisfaction; Self-efficacy; education in economically disadvantaged area
**Abstract:**	The unequal distribution of educational resources between regions with varying levels of development is a global issue. Teachers in economically disadvantaged areas often have low work engagement due to limited resources and personal development opportunities, leading to negative attitudes that harm educational progress. Improving self-efficacy and job satisfaction are essential strategies to enhance work engagement, but these have not been fully explored in underdeveloped regions. To address this gap, we collected 472 questionnaires from rural primary school teachers, who completed a 5-point scale survey. A path model was used to analyze the direct and indirect effects of self-efficacy and job satisfaction on work engagement. The findings indicate that self-efficacy indirectly influences work engagement, with job satisfaction serving as a crucial mediator. Our study concludes that increasing self-efficacy in teachers from economically disadvantaged areas boosts job satisfaction, which subsequently enhances their work engagement.
**Order of Authors:**	Hongping Zhou
	Shi Qi Xu
	Dong-Hwa Chung
	De Xin Dang
**Additional Information:**	
**Question**	**Response**
**Financial Disclosure**<br><br>Enter a financial disclosure statement that describes the sources of funding for the work included in this submission. Review the submission guidelines for detailed requirements. View published research articles from *PLOS ONE* for specific examples.<br><br>This statement is required for submission and **will appear in the published article** if the submission is accepted. Please make sure it is accurate.	Yes

**Funded studies**<br>Enter a statement with the following details:<br>• Initials of the authors who received each award<br>• Grant numbers awarded to each author<br>• The full name of each funder<br>• URL of each funder website<br>• Did the sponsors or funders play any role in the study design, data collection and analysis, decision to publish, or preparation of the manuscript?<br><br>Did you receive funding for this work?	
Please add funding details.<br>as follow-up to "**Financial Disclosure**<br><br>Enter a financial disclosure statement that describes the sources of funding for the work included in this submission. Review the submission guidelines for detailed requirements. View published research articles from *PLOS ONE* for specific examples.<br><br>This statement is required for submission and **will appear in the published article** if the submission is accepted. Please make sure it is accurate.<br><br>**Funded studies**<br>Enter a statement with the following details:<br>• Initials of the authors who received each award<br>• Grant numbers awarded to each author<br>• The full name of each funder<br>• URL of each funder website<br>• Did the sponsors or funders play any role in the study design, data collection and analysis, decision to publish, or preparation of the manuscript?<br><br>Did you receive funding for this work?"	This study was supported by a grant from the National Social Science Fund of China (Research on the Occupational Ecological Dilemma and Collaborative Governance of Rural Physical Education Teachers in the New Era; agreement number 21BTY101).
Please select the country of your main research funder (please select carefully as in some cases this is used in fee calculation).	CHINA - CN

as follow-up to "**Financial Disclosure**<br><br>Enter a financial disclosure statement that describes the sources of funding for the work included in this submission. Review the submission guidelines for detailed requirements. View published research articles from *PLOS ONE* for specific examples.<br><br>This statement is required for submission and **will appear in the published article** if the submission is accepted. Please make sure it is accurate.<br><br>**Funded studies**<br>Enter a statement with the following details:<br>• Initials of the authors who received each award<br>• Grant numbers awarded to each author<br>• The full name of each funder<br>• URL of each funder website<br>• Did the sponsors or funders play any role in the study design, data collection and analysis, decision to publish, or preparation of the manuscript?<br><br>Did you receive funding for this work?"	
**Competing Interests**<br><br>Use the instructions below to enter a competing interest statement for this submission. On behalf of all authors, disclose any competing interests that could be perceived to bias this work—acknowledging all financial support and any other relevant financial or non-financial competing interests.<br><br>This statement is required for submission and **will appear in the published article** if the submission is accepted. Please make sure it is accurate and that any funding sources listed in your Funding Information later in the submission form are also declared in your Financial Disclosure statement.	All the authors declare that they have no conflict of interest.

Powered by Editorial Manager® and ProduXion Manager® from Aries Systems Corporation

View published research articles from *PLOS ONE* for specific examples.<br><br>**NO authors have competing interests**<br><br>Enter: *The authors have declared that no competing interests exist.*<br><br>**Authors with competing interests**<br><br>Enter competing interest details beginning with this statement:<br><br>*I have read the journal's policy and the authors of this manuscript have the following competing interests: [insert competing interests here]*<br><br>* typeset	
**Ethics Statement**<br><br>Enter an ethics statement for this submission. This statement is required if the study involved:<br><br>• Human participants<br>• Human specimens or tissue<br>• Vertebrate animals or cephalopods<br>• Vertebrate embryos or tissues<br>• Field research<br><br>Write "N/A" if the submission does not require an ethics statement.<br><br>General guidance is provided below. Consult the submission guidelines for detailed instructions. **Make sure that all information entered here is included in the Methods section of the manuscript.**	The procedure and protocol employed in this study were approved by the ethics committee of Hubei University of Arts and Science (Xiangyang, China).

Powered by Editorial Manager® and ProduXion Manager® from Aries Systems Corporation

**Format for specific study types**<br><br>**Human Subject Research (involving human participants and/or tissue)**<br>• Give the name of the institutional review board or ethics committee that approved the study<br>• Include the approval number and/or a statement indicating approval of this research<br>• Indicate the form of consent obtained (written/oral) or the reason that consent was not obtained (e.g. the data were analyzed anonymously)<br><br>**Animal Research (involving vertebrate animals, embryos or tissues)**<br>• Provide the name of the Institutional Animal Care and Use Committee (IACUC) or other relevant ethics board that reviewed the study protocol, and indicate whether they approved this research or granted a formal waiver of ethical approval<br>• Include an approval number if one was obtained<br>• If the study involved *non-human primates*, add *additional details* about animal welfare and steps taken to ameliorate suffering<br>• If anesthesia, euthanasia, or any kind of animal sacrifice is part of the study, include briefly which substances and/or methods were applied<br><br>**Field Research**<br><br>Include the following details if this study involves the collection of plant, animal, or other materials from a natural setting:<br>• Field permit number<br>• Name of the institution or relevant body that granted permission	
**Data Availability**<br><br>Authors are required to make all data underlying the findings described fully available, without restriction, and from the time of publication. PLOS allows rare exceptions to address legal and ethical concerns. See the PLOS Data Policy and FAQ for detailed information.	Yes - all data are fully available without restriction

A Data Availability Statement describing where the data can be found is required at submission. Your answers to this question constitute the Data Availability Statement and **will be published in the article**, if accepted.<br><br>**Important:** Stating 'data available on request from the author' is not sufficient. If your data are only available upon request, select 'No' for the first question and explain your exceptional situation in the text box.<br><br>Do the authors confirm that all data underlying the findings described in their manuscript are fully available without restriction?	
**Describe where the data may be found in full sentences. If you are copying our sample text, replace any instances of XXX with the appropriate details.**<br><br>• If the data are **held or will be held in a public repository**, include URLs, accession numbers or DOIs. If this information will only be available after acceptance, indicate this by ticking the box below. For example: *All XXX files are available from the XXX database (accession number(s) XXX, XXX.*).<br>• If the data are all contained **within the manuscript and/or Supporting Information files**, enter the following: *All relevant data are within the manuscript and its Supporting Information files.*<br>• If neither of these applies but you are able to provide **details of access elsewhere**, with or without limitations, please do so. For example:<br><br>*Data cannot be shared publicly because of [XXX]. Data are available from the XXX Institutional Data Access / Ethics Committee (contact via XXX) for researchers who meet the criteria for access to confidential data.*<br><br>*The data underlying the results presented in the study are available from (include the name of the third party*	The data that support the findings of this study are available from the corresponding author, Shi Qi Xu (xsq941214@naver.com), upon reasonable request.

and contact information or URL).<br>• This text is appropriate if the data are owned by a third party and authors do not have permission to share the data.<br><br>* typeset	
Additional data availability information:	

Powered by Editorial Manager® and ProduXion Manager® from Aries Systems Corporation

Manuscript Click here to access/download;Manuscript;24.09.25.Rural teacher manuscript anonymous(1).docx

Running title: work engagement & rural teacher

Job satisfaction mediates effect of self-efficacy on work engagement for teachers in economically disadvantaged areas

Hongping Zhou[1], Shi Qi Xu[1,#], Dong-Hwa Chung[4], De Xin Dang[2,3,#]

[1]Institute of Physical Education, Hubei University of Arts and Science, Xiangyang 441053, China

[2]Department of Endocrinology, Xiangyang Central Hospital, Affiliated Hospital of Hubei University of Arts and Science, Hubei University of Arts and Science, Xiangyang 441021, China

[3]The First Affiliated Hospital of Guangzhou Medical University, Guangzhou Medical University, Guangzhou 510120, China

[4]Department of Sports Convergence Technology, Sangmyung University, Seoul 03016, South Korea

*Corresponding author: DX Dang (Email: ddx2533387852@gmail.com); SQ Xu (E-mail: xsq941214@naver.com)

Abstract

The unequal distribution of educational resources between regions with varying levels of development is a global issue. Teachers in economically disadvantaged areas often have low work engagement due to limited resources and personal development opportunities, leading to negative attitudes that harm educational progress. Improving self-efficacy and job satisfaction are essential strategies to enhance work engagement, but these have not been fully explored in underdeveloped regions. To address this gap, we collected 472 questionnaires from rural primary school teachers, who completed a 5-point scale survey. A path model was used to analyze the direct and indirect effects of self-efficacy and job satisfaction on work engagement. The findings indicate that self-efficacy indirectly influences work engagement, with job satisfaction serving as a crucial mediator. Our study concludes that increasing self-efficacy in teachers from economically disadvantaged areas boosts

job satisfaction, which subsequently enhances their work engagement.

Keywords: rural teacher, work engagement, job satisfaction, self-efficacy, education in economically disadvantaged area

1. Introduction

The imbalance in educational resources between regions with varying levels of development is a global issue, particularly in developing countries and regions [1]. Compared to developed regions, teachers in economically disadvantaged areas face a shortage of educational resources and equipment. This results in limited opportunities for professional growth and a poorer quality of life, leading to lower work fulfillment and work engagement [2]. Consequently, these teachers often exhibit negative and perfunctory attitudes, further hindering the development of education in these economically disadvantaged areas [3]. Therefore, increasing the work engagement of rural teachers has become a focus for many scholars.

Studies have reported that enhancing self-efficacy and job satisfaction are effective measures to increase work engagement. Self-efficacy reflects teachers' confidence in using effective methods to facilitate student learning, manage classrooms, and promote student development during the educational process [4]. Moreover, job satisfaction represents the sense of accomplishment and satisfaction derive from the work [5].

Multiple studies have demonstrated that higher self-efficacy can increase teachers' work engagement [6-8], thereby creating a positive learning environment [9]. Self-efficacy indicates teachers' confidence in conducting educational practices [10]. Teachers who believe in their educational abilities are better equipped to face the various roles and challenges within the educational practice [11,12]. This confidence makes them more willing to improve educational methods and actively participate in educational activities, thus increasing their work engagement [13,14].

Job satisfaction also plays a crucial role in enhancing work engagement [15-17]. Increasing job satisfaction generates positive effects on work engagemen [18]. Oubibi et al. [19] reported results obtained in

educational environments, demonstrating a significant positive correlation between job satisfaction and work engagement. Teachers with high job satisfaction are more enthusiastic about their work, leading to increased investment of time and energy into the educational process and student services [20].

Therefore, enhancing self-efficacy and job satisfaction can effectively improve work engagement among teachers. However, limited studies have explored the role of job satisfaction and self-efficacy in improving work engagement for teachers in economically disadvantaged areas. Focusing on areas with scarce educational resources and addressing the work engagement of teachers in these regions is a crucial step towards narrowing the educational resource gap. Improving the work engagement of rural teachers will benefit the development of education in economically disadvantaged areas. Therefore, the purpose of this study was to investigate the impact of self-efficacy and job satisfaction on teachers' work engagement, providing new strategies for the development of education in economically disadvantaged areas.

2. Literature review and research hypothesis

Low work engagement among teachers in economically disadvantaged areas is a significant obstacle to the development of education in these regions. Studies have demonstrated that work engagement is crucial for enhancing career satisfaction and happiness among teachers, which in turn stimulates student interest in learning, improves studying performance, and optimizes educational outcome [21-23]. Therefore, measures should be implemented to increase work engagement among teachers in economically disadvantaged areas. This will help these teachers exhibit higher levels of professionalism and enthusiasm, enabling them to face work-related challenges with positive emotions, persist in improving educational methods, and ultimately enhance educational outcomes. Enhancing self-efficacy and job satisfaction are effective measures to increase work engagement among these teachers.

In the field of education, self-efficacy reflects teachers' confidence in using effective methods to facilitate student learning, manage classrooms, and promote student development during the educational process [4].

The degree of a teacher's confidence not only affects their teaching attitude and methods but also determines their persistence and effort when facing challenges. Teachers with high self-efficacy are usually enthusiastic about teaching, enjoy the teaching process, and maintain high professional happiness with a healthy mindset [24]. They are also good at self-assessment, continually improving based on feedback, thereby optimizing teaching quality.

Furthermore, job satisfaction represents the sense of accomplishment and fulfillment derive from their work [5], which is a decisive factor affecting teachers' attitudes and performance [25]. Declining job satisfaction can reduce teachers' willingness to work, thereby impairing student performance [26]. It has been proven that measures to increase job satisfaction among teachers can enhance their positivity, make teacher-student relationships more harmonious, and ultimately achieve better educational outcomes [27,28].

By enhancing self-efficacy, teachers in economically disadvantaged areas may achieve higher job satisfaction, which can lead to greater work engagement and benefit the development of education in these areas. However, limited studies have investigated measures to increase work engagement among teachers in economically disadvantaged areas. Further development of education in these regions will benefit from increasing work engagement among teachers. Therefore, the objective of this study was to explore the role of job satisfaction and self-efficacy in improving work engagement in economically disadvantaged areas, contributing to providing new strategies for the development of education in these regions.

2.1 Self-efficacy

The theory of self-efficacy was initially proposed by Bandura in 1982, highlighting that motivation and behavior are influenced not only by external factors but also by individuals' confidence [29]. In educational settings, self-efficacy refers to teachers' confidence in successfully fulfilling their educational roles and overcoming challenges [30,31]. Self-efficacy is essential for teachers to achieve satisfactory educational outcomes [32,33].

Individuals with high self-efficacy tend to proactively seek challenging roles and are willing to invest more time and effort to achieve their goals, persevering even when faced with setbacks [14]. In educational environments, teachers with high self-efficacy experience have better mental health, exhibit lower burnout and fatigue [34,35], collaborate effectively with colleagues to achieve common educational goals [36], and importantly, report higher job satisfaction [37], ultimately enhancing students' studying performance [10,38].

Self-efficacy consists of three dimensions: instructional strategies, classroom management, and student engagement [39].

Instructional strategies emphasize teachers' beliefs in their ability to choose and implement effective teaching strategies and methods to achieve educational objectives, effectively impart knowledge to students, and promote their learning and understanding. These strategies include creating engaging and interactive classroom environments that stimulate students' interest, cater to their individual learning needs, and facilitate deeper understanding [40]. Effective instructional strategies are closely linked to students' studying performance, as interactive and targeted teaching methods create engaging learning experiences and student-centered classrooms adaptable to diverse educational environments and student needs [41,42].

Classroom management is critical for effectively carrying out educational activities [39,43]. Teachers' confidence in their ability to establish and enforce classroom rules, manage student behavior effectively, and create a conducive learning environment helps maintain focus on educational content [44], increase student engagement [45], reduce teacher stress and fatigue, and thereby enhance job satisfaction [46,47].

Student engagement reflects teachers' ability and confidence in stimulating and maintaining students' involvement [39]. By designing engaging lesson content, implementing interactive teaching methods, and fostering positive teacher-student interactions, teachers can enhance student motivation and promote deeper understanding for studying content [48].

2.2 Job satisfaction

Job satisfaction reflects an individual's overall attitude and emotional response towards various aspects of their work, such as salary, promotion opportunities, colleagues, supervision, and work content [49-52]. In educational environments, job satisfaction is a critical factor influencing teachers' attitudes and performance [25]. Decreased job satisfaction among teachers can lead to increased burnout and turnover intentions, directly impacting students' studying performance [27,53]. Moreover, it can contribute to psychological issues such as depression, anxiety, and loneliness among teachers [28,54-57]. Measures aimed at enhancing teachers' job satisfaction can optimize their educational performance [26,58], help establish effective educational environments, improve educational quality [55], foster collaborative relationships among colleagues [54], and ultimately enhance students' studying performance and experience [53].

Teachers' job satisfaction is influenced by factors such as intrinsic rewards of teaching (job recognition and social status), intrinsic rewards of teaching (course teaching), and school-based factors (work environment and welfare treatment) [59,60].

Course teaching involves the time, effort, and resources invest in designing, implementing, and evaluating educational activities. Higher satisfaction with course teaching indicates that teachers are content with the preparation and implementation processes. Teachers satisfied with course teaching can design appropriate educational content that aligns with educational objectives and student needs, structure effective classroom environments, facilitate organized educational delivery, and stimulate student interest and proactive learning [48,61].

Welfare treatment encompasses teachers' overall economic, security, and career development benefits, including salary, insurance, retirement rewards, paid vacation, professional training, and promotion opportunities [62]. These benefits directly impact teachers' economic stability, professional security, and career advancement. Teachers satisfied with welfare treatment demonstrate high efficacy in classroom management, which correlates with higher studying performance among students [63]. Conversely, inadequate welfare can impair teachers' educational performance [64].

Work environment encompasses teachers' perceptions and evaluations of school culture, educational resources, and administrative conditions [65]. A supportive work environment provides necessary resources and support, enhances teachers' sense of belonging and professional identity [27,66], fosters respect and recognition for their work, and motivates continuous improvement in professional skills and educational abilities [67].

Job recognition refers to how much teachers feel valued, respected, and supported by leaders and colleagues, including leaders' appreciation for teachers' efforts, collegial relationships, and recognition of professional competence. Feeling supported by school leaders increases teachers' sense of pride and accomplishment, positively influencing their performance and satisfaction [68]. Similarly, recognition from colleagues boosts confidence, fosters a positive work environment, and encourages teachers to excel in their teaching roles [69].

Social status measures the level of respect and recognition receive for teachers' professional contributions from various societal sectors, including parents, government agencies, media, community organizations, and the public. This recognition encompasses appreciation for teachers' professional skills, educational achievements, and their role in society and the community [70]. Studies indicate that positive social status in teaching careers correlates with increased work engagement, educational outcomes, professional self-esteem, and feelings of accomplishment [27,71]. When teachers feel recognized socially, they gain confidence and a sense of purpose in their work. Social status not only enhances teachers' enthusiasm for their work but also motivates them to invest time and effort into implementing innovative teaching methods to improve educational quality [53].

2.3 Work engagement

Work engagement encompasses the commitment to work roles, pursuit of work objectives, and emotional attachment and identification with one's work. High work engagement reflects wholehearted dedication to

work tasks, intense focus, and reduced fatigue, contributing to greater personal happiness [72,73]. It is a persistent cognitive state, not tied to specific events, individuals, behaviors, or goals [74].

Work engagement, as a positive work attitude, comprises three dimensions: vigor, dedication, and absorption [74,75].

Vigor reflects viewing one's career as an important goal and pursuing it with abundant energy and psychological resilience, even in the face of challenges [76]. Vigor is not only a positive physiological state that enhances work performance [77], but also increases professional happiness [78,79]. Individuals with high vigor are better able to collaborate with colleagues, overcome challenges collectively, and achieve organizational objectives [80].

Dedication is an emotional state characterized by enthusiasm, wholeheartedness, and seeing work as important and challenging [81]. Research indicates that individuals high in dedication typically exhibit high work performance [76], job satisfaction [82], organizational loyalty [83], and teamwork [84]. They invest significant time and effort into their work, derive satisfaction and a sense of accomplishment from it, and their enthusiasm positively influences colleagues, fostering teamwork and improving overall work atmosphere.

Absorption is a heightened state of focus where individuals become fully engrossed in their work roles, experiencing happiness and a flow state where time seems to fly by unnoticed [76,81]. Those with high absorption are deeply immersed in their tasks, maintaining high concentration and productivity, thereby enhancing work efficiency and quality [85]. This absorption leads to a sense of accomplishment and satisfaction [86].

2.4 Self-efficacy & work engagement

Self-efficacy for teachers involves their confidence in achieving positive educational outcomes during educational practices within their own teaching environments [10]. Teachers who trust in their educational abilities and influence are better equipped to handle various roles and challenges in the educational process.

This belief motivates them to invest time and effort into improving their teaching methods, actively participating in educational activities, persistently enhancing their professional skills and educational performance, thereby increasing their work engagement. Thus, self-efficacy forms an essential precondition for work engagement [87].

Teachers with high self-efficacy are more likely to implement innovative educational practices, possess better classroom management skills, and engage more effectively in professional development [11,12]. They maintain a positive attitude when facing pressure and challenges in education, devising effective strategies to handle stress and minimizing its negative impact on their work, thereby sustaining higher levels of work engagement [6]. Research indicates that teachers with high self-efficacy are more motivated in their work, investing greater emotional and mental energy, resulting in higher work engagement [13]. Therefore, higher self-efficacy among teachers contributes to increased work engagement [6-8], thereby creating a more positive learning environment [9].

Thus, we hypothesize that an increase in self-efficacy will lead to increased work engagement among teachers in economically disadvantaged areas (Hypothesis 1). If Hypothesis 1 is supported, it suggests that self-efficacy directly influences work engagement among teachers in economically disadvantaged areas.

2.5 Self-efficacy & job satisfaction

Furthermore, self-efficacy among teachers is closely associated with job satisfaction [88-90]. As previously discussed, self-efficacy refers to teachers' confidence in their ability to plan, organize, and achieve educational objectives in their teaching contexts. Teachers with higher self-efficacy believe in their educational capabilities, enabling them to adapt to different educational environments and meet students' needs, thereby experiencing a sense of accomplishment and satisfaction, which increases job satisfaction. Research indicates that teachers with high self-efficacy derive greater satisfaction from their educational activities [91,92] and report lower feelings of burnout [93].

Teachers with higher self-efficacy feel more in control of their work environment and outcomes, enhancing their ability to effectively manage stress and mitigate its impact. In contrast, teachers with low self-efficacy may perceive difficulties in classroom management and meeting student demands, leading to disruptions and ongoing teaching challenges that erode their confidence. This perception can increase their stress levels and hinder effective problem-solving strategies [94].

Therefore, we hypothesize that job satisfaction among teachers in economically disadvantaged areas will increase with higher levels of self-efficacy (Hypothesis 2a).

2.6 Job satisfaction & work engagement

Job satisfaction also significantly impacts work engagement [16,17]. Teachers who experience high job satisfaction are more likely to be enthusiastic about their work, investing greater time and effort in teaching and supporting students [20]. Numerous studies have consistently shown a positive relationship between job satisfaction and work engagement among teachers [95-102], as teachers with high job satisfaction derive internal motivation and purpose from their work [93].

Therefore, we hypothesize that work engagement among teachers in economically disadvantaged areas will increase with higher levels of job satisfaction (Hypothesis 2b). If both Hypothesis 2a and 2b are supported, it suggests that self-efficacy indirectly influences work engagement among teachers in economically disadvantaged areas, with job satisfaction playing a crucial mediating role in this relationship (Hypothesis 2).

2.7 Conceptual framework

The purpose of this study is to investigate the roles of job satisfaction and self-efficacy in enhancing work engagement among teachers in economically disadvantaged areas. Based on the theoretical assumptions discussed, this study has developed a research model (Fig. 1).

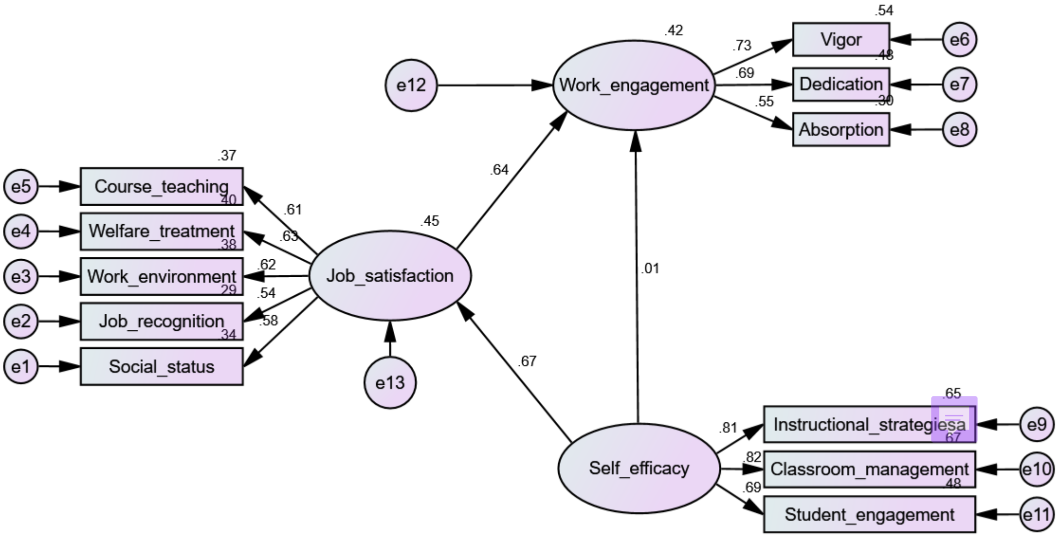

Fig. 1. Path analysis (calculated using AMOS).

Fitting degree of model: Cmin/df, 2.175; GFI, 0.969; Adjusted GFI, 0.950; Root mean standard error of approximation, 0.050; Tucker-Lewis index, 0.956; Normed fit index, 0.941; IFI, 0.967; CFI, 0.967; SRMR, 0.036

3. Materials and methods

The procedure and protocol employed in this study were approved by the ethics committee of Hubei University of Arts and Science (Xiangyang, China).

3.1 Survey design and questionnaire

The questionnaire used in this study was administered and web-programmed by Wen Juan Xing (Changsha Ranxing IT Ltd.) and distributed via WeChat and email. It consisted of closed-ended questions divided into four parts (Table 1).

Table 1. Compositions of questionnaire

Variables	Questions
Sociodemographic variables, questions, and scales used in the questionnaires	
Age (years) (1: 20-30 years old; 2: 31-40	How old are you?

years old; 3: 41-50 years old; 4: 51 years and above)	
Gender (1: male; 2: female)	What is your gender?
Educational background (1: college degree; 2: bachelor degree; 3: master degree; 4: doctoral degree)	What is your educational background?
Professional title: (1: junior; 2: medium; 3: senior)	What is your professional title?
Contract type (1: full-time; 2: part-time)	What is your contract type?
Weekly teaching hours (1: less than 10 times; 2: 10-15 times; 3: 16-20 times; 4: 21 times and above)	How many hours of class do you have in a week? (without competition organization, extracurricular sports activities guidance, and extracurricular sports training, etc.)
Monthly salary (1: 2000-3000 yuan; 2: 3001-4000 yuan; 3: 4001-5000 yuan; 4: 5001 yuan and above)	How much money do you earn in a week?
Variables, questions, and scales of self-efficacy used in the questionnaires (Tschannen-Moran and Hoy, 2001)	
Instructional strategies (1-5: very dissatisfied – very satisfied)	1. To what extent can you use various assessment strategies?<br>2. To what extent can you provide an alternative explanation or example when students feel confused?<br>3. To what extent can you encourage your students to use self-regulated learning strategies?<br>4. How capable are you of implementing alternative strategies in the classroom?<br>5. To what extent can you inform students about the importance and usefulness of self-directed learning strategies?<br>6. To what extent can you teach your students how to use and apply different self-regulated learning strategies?<br>7. To what extent can you measure students' understanding of the content you teach?<br>8. To what extent can you provide appropriate challenges for highly capable students?
Classroom management (1-5: very dissatisfied – very satisfied)	1. To what extent can you control disruptive behavior in the classroom?<br>2. What can you do to ensure that children follow classroom rules?<br>3. What can you do to calm down a disruptive or noisy student?<br>4. To what extent can you establish a classroom management system for each group of students?<br>5. To what extent can you avoid disruptive behavior from a few students disrupting the entire class?<br>6. How can you respond to challenging students?<br>7. To what extent can you clearly communicate your expectations for student behavior?<br>8. To what extent can you establish routines to keep activities running smoothly?
Student engagement (1-5: very dissatisfied	1. What can you do to make students believe they can do their

– very satisfied)	homework well?<br>2. What can you do to help your students value learning?<br>3. What can you do to motivate students who are not interested in academics?<br>4. To what extent can you help families support their children in achieving good grades at school?<br>5. What can you do to improve understanding for students who are failing?<br>6. What can you do to help your students think critically?<br>7. What can you do to foster creativity in students?<br>8. How much can you do to help the most difficult students to get along with?
Variables, questions, and scales of job satisfaction used in the questionnaires (Chen, 2023; Bian, 2023; Huang, 2024)	
Course teaching (1-5: very dissatisfied – very satisfied)	1. How much do you think teaching well impacts your career development?<br>2. Are you satisfied with the time and effort you invest in teaching?<br>3. Are you satisfied with the curriculum system?<br>4. Are you satisfied with the workload of teaching?<br>5. Are you satisfied with the preparation time and frequency?<br>6. Are you satisfied with how students respond in class?
Welfare treatment (1-5: very dissatisfied – very satisfied)	1. Are you satisfied with the salary level of physical education teachers at this school?<br>2. Are you satisfied with the opportunities for career advancement for physical education teachers at this school?<br>3. Are you satisfied with the fairness and reasonableness of the system for evaluating professional titles for physical education teachers at this school?
Work environment (1-5: very dissatisfied – very satisfied)	1. Are you satisfied with the campus culture atmosphere?<br>2. Are you satisfied with the teaching environment at the school?<br>3. Are you satisfied with the office conditions at the school?
Job recognition (1-5: very dissatisfied – very satisfied)	1. How much importance do you think school leadership places on physical education?<br>2. Are you satisfied with your relationship with school leadership?<br>3. How much recognition do you feel your colleagues give you?<br>4. Are you satisfied with how well you cooperate and communicate with your colleagues?
Social status (1-5: very dissatisfied – very satisfied)	1. How do you perceive the societal evaluation of rural physical education teachers?<br>2. Are you satisfied with the social recognition of the status of rural physical education teachers?<br>3. Are you satisfied with the social status of rural physical education teachers?
Variables, questions, and scales of work engagement used in the questionnaires (Schaufeli et al., 2002)	

Vigor (1-5: very dissatisfied – very satisfied)	1. I always feel energetic while working.<br>2. I often feel strong and full of vitality in my work.<br>3. When I wake up in the morning, I look forward to going to work.<br>4. I can work for long periods of time without feeling tired.<br>5. I have strong psychological resilience in my work.<br>6. Even when things are not going well in my work, I always persevere.
Dedication (1-5: very dissatisfied – very satisfied)	1. I believe that my work is meaningful and purposeful.<br>2. I am passionate about my work.<br>3. I feel that my work motivates me.<br>4. I am proud to be a rural physical education teacher.<br>5. I find the work of a rural physical education teacher challenging.
Absorption (1-5: very dissatisfied – very satisfied)	1. Time passes quickly when I'm working.<br>2. I become completely absorbed in my work.<br>3. I feel happy when I work hard.<br>4. I am deeply engaged in the work of a rural physical education teacher.<br>5. I always feel excited when I'm working.<br>6. It's always hard for me to stop working.

Section 1 comprised 7 questions aimed at gathering socio-demographic information from respondents, including age, gender, educational background, professional title, contract type, weekly teaching hours, and monthly salary.

Section 2 comprised 24 questions designed refer to the Teachers' Sense of Efficacy Scale proposed by Tschannen-Moran and Hoy [38] to assess self-efficacy across three variables: instructional strategies (8 questions), classroom management (8 questions), and student engagement (8 questions). Responses were recorded on a 5-point scale.

Section 3 comprised 19 questions designed refer to the questionnaires designed by Chen and Sun [103], Bian [104], and Huang [105] to assess job satisfaction across five variables: course teaching (6 questions), welfare treatment (3 questions), job recognition (4 questions), social status (3 questions), and work environment (3 questions). Responses were also measured on a 5-point scale.

Section 4 comprised 17 questions designed refer to the Utrecht Work Engagement Scale proposed by Schaufeli et al. [74] to assess work engagement across three variables: vigor (6 questions), absorption (6 questions), and dedication (5 questions). Responses were assessed using a 5-point scale.

3.2 Data collection and analysis

The questionnaire was distributed to frontline Physical Education Teachers from 138 primary schools in Hubei Province, China, using convenient sampling [106]. Respondents were approached face-to-face during school visits. Out of 526 responses received, 54 were deemed unusable, leaving 472 questionnaires for analysis. Prior to participation, all respondents were informed about the study's purpose and provided written informed consent. The collection period started on 21/03/2022, and was completed on 28/05/2023.

3.3 Statistical analysis

Data were analyzed using SPSS (version 26.0). Frequency analysis was employed to examine respondents' general characteristics. The validity and reliability of the questionnaire were assessed through confirmatory factor analysis and Cronbach's alpha [107]. Convergent validity was considered satisfactory for multi-item scales if the Average Variance Extracted value exceeded 0.5 and Composite Reliability exceeded 0.8 [108].

The research hypotheses were tested using the structural equation modeling method conducted with AMOS. We followed the two-stage approach proposed by Anderson and Gerbing [109]: the first stage involved confirmatory factor analysis to estimate the items in the research model, and the second stage examined structural relationships among constructs to test research hypotheses. Statistical significance was set at $P < 0.05$. Model fit indices including CMIN/DF, SRMR, GFI, AGFI, NFI, IFI, TLI, CFI, and RMSEA were assessed against thresholds of <3, <0.05, >0.9, >0.9, >0.9, >0.9, >0.9, >0.9, and <0.08, respectively, to determine model adequacy [110].

4. Results

4.1 Demographic characteristics of respondents

A total of 472 respondents participated in this investigation, with 37.92% being male and 62.08% female.

Most respondents were engaged in full-time contracts (98.52%), while the remaining 1.48% were on part-time contracts. Regarding age distribution, 19.49% were aged 20-30, 26.91% were aged 31-40, 38.35% were aged 41-50, and 15.25% were older than 51 years. Educationally, more than half of the respondents held a bachelor’s degree (88.35%), followed by those with a college degree (9.32%) and a master’s degree (2.33%). In terms of professional title, 56.78% held a medium-level title, 14.62% held a junior-level title, and 28.60% held a senior-level title. Regarding teaching workload, 7.42% of respondents had fewer than 10 lessons per week, 31.14% had 10-15 lessons per week, 41.10% had 16-20 lessons per week, and 20.34% had more than 21 lessons per week. Concerning salary, 3.39% earned 2000-3000 yuan per month, 31.35% earned 3001-4000 yuan per month, 48.10% earned 4001-5000 yuan per month, and 17.16% earned more than 5000 yuan per month (Fig. 2).

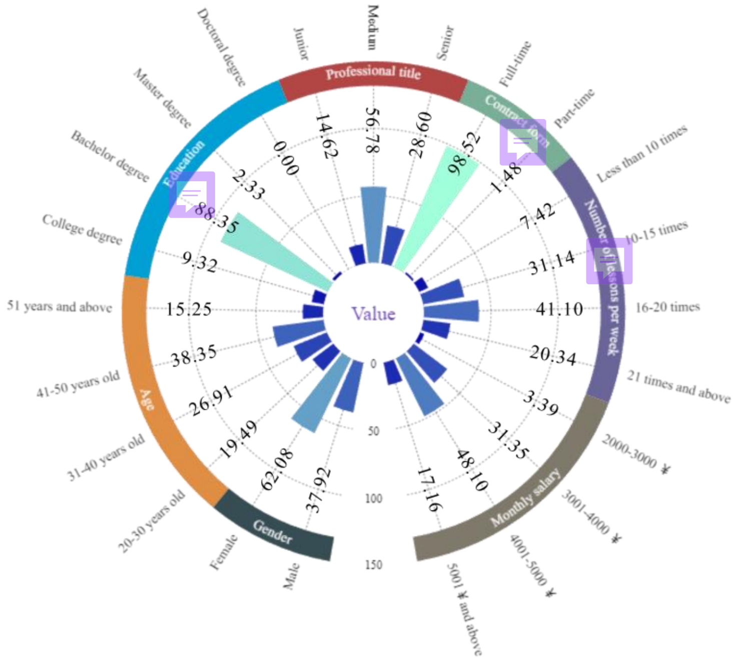

Fig. 2. Demographic characteristics of respondents

4.2 Results of factor analysis

Based on the results of the factor analysis of self-efficacy (Supplementary Fig. 1), job satisfaction (Supplementary Fig. 2), and work engagement (Supplementary Fig. 3), no questions exhibited low factor loading values. The factor analysis across these three measures was deemed reasonable based on the

Cronbach's α coefficients for all factors. Additionally, the results of convergent and discriminant validity statistics showed that all average variance extracted and composite reliability values for the multi-item scales were greater than 0.5 and 0.8, respectively. This indicates a sufficient level of convergent validity for the measurement model.

4.3 Model parameters

The parameters confirmed that the proposed measurement model fit the data well: Cmin/df = 2.175, GFI = 0.969, adjusted GFI = 0.950, RMSEA = 0.050, TLI = 0.956, NFI = 0.941, SRMR = 0.036, IFI = 0.967, and CFI = 0.967 (Fig. 3).

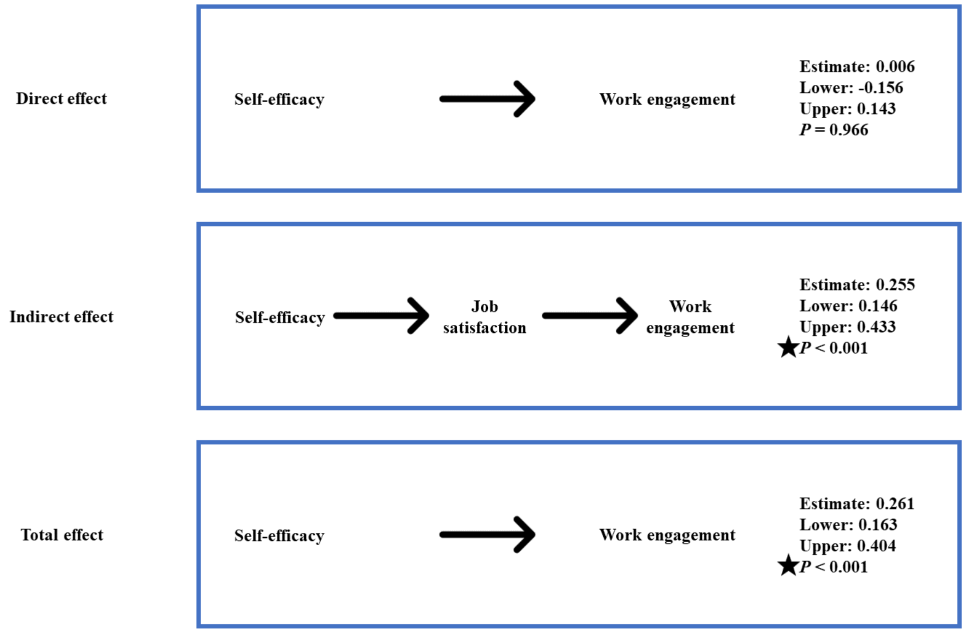

Fig. 3. Results of structural equation modeling

4.4 Indirect and total effects

The results of structural equation modeling indicated that self-efficacy indirectly influences work engagement, with job satisfaction playing a crucial mediating role in this relationship (Fig. 4).

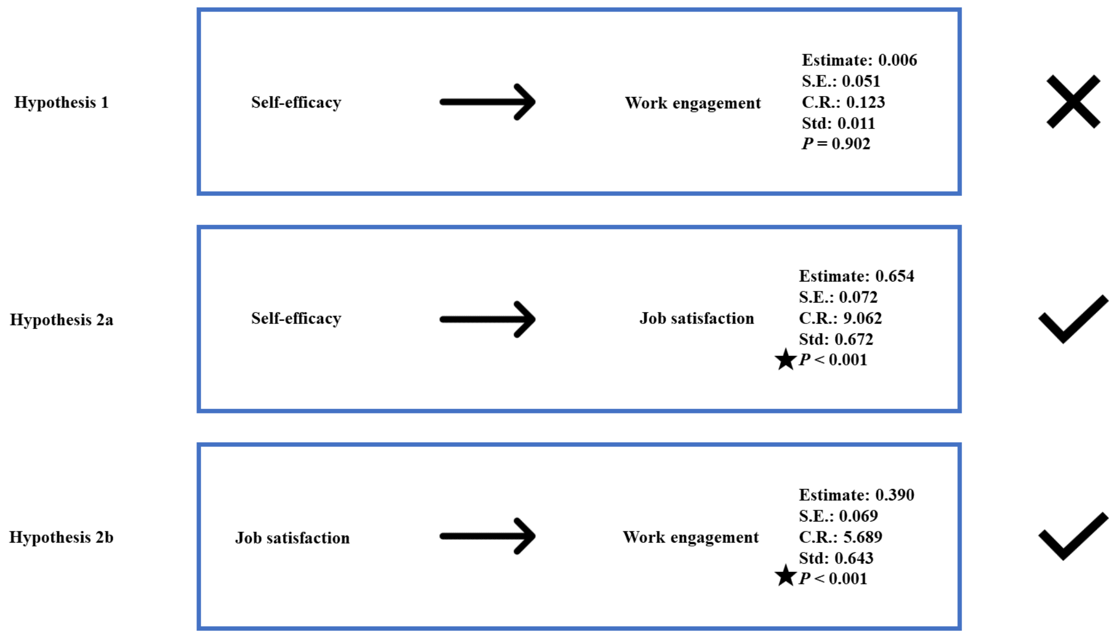

Fig. 4. Summary of support for hypothesis

4.5 Hypothesis testing

The results of hypothesis testing indicated that self-efficacy has a significant direct effect on job satisfaction (self-efficacy → job satisfaction = 0.654, $P < 0.001$), and job satisfaction has a significant direct effect on work engagement (job satisfaction → work engagement = 0.390, $P < 0.001$). These findings supported hypotheses 2a and 2b. However, self-efficacy has no significant direct effect on work engagement, leading to the rejection of hypothesis 1 (Fig. 1).

5. Discussion

This study explores the role of job satisfaction and self-efficacy in enhancing work engagement for teachers in economically disadvantaged areas. We found that self-efficacy indirectly influences work engagement, with job satisfaction playing a crucial mediating role in this relationship.

Our results support the notion that self-efficacy has a substantial impact on job satisfaction among teachers in economically disadvantaged areas. Ortan et al. [111]. similarly observed that increasing teachers'

self-efficacy enhances job satisfaction. Türkoglu et al. [112] also identified a significant positive correlation between self-efficacy and job satisfaction. Self-efficacy reflects teachers' confidence in overcoming challenges during the educational process [38]. Teachers with high self-efficacy are more inclined to complete educational tasks effectively, viewing challenges as manageable [39]. Consequently, these teachers are less likely to experience frustration or disappointment, finding fulfillment in their work and thereby increasing their job satisfaction. Therefore, enhancing self-efficacy among teachers in economically disadvantaged areas can further elevate their job satisfaction, validating hypothesis 2a proposed in this study.

Furthermore, we identified that job satisfaction significantly predicts work engagement. Increased job satisfaction fosters positive effects on work engagement [18]. Oubibi et al. [113] also reported a significant positive correlation between job satisfaction and work engagement in educational environments. Teachers with higher job satisfaction tend to invest more time and effort in their educational roles [52,114], demonstrating higher levels of vigor, dedication, and absorption—essential components of work engagement [74]. Therefore, enhancing teachers' job satisfaction is crucial for improving their work engagement, hypothesis 2b proposed in this study has been validated.

The validation of hypotheses 2a and 2b in this study indicates that self-efficacy indirectly influences work engagement, with job satisfaction playing a crucial mediating role in this relationship. Enhancing self-efficacy among teachers in economically disadvantaged areas can effectively increase their job satisfaction, thereby improving their work engagement. Specifically, increased self-efficacy enables teachers to approach educational activities with confidence, fostering positive attitudes and behaviors at work. However, our findings suggest that confidence alone, as hypothesized in hypothesis 1, may not be sufficient to enhance work engagement. While self-efficacy positively impacts work engagement, job satisfaction is essential for achieving this outcome. As noted by Fathi and Derakhshan [34], teachers with high self-efficacy maintain a positive emotional state towards their work, which enhances job satisfaction and subsequently facilitates work engagement. Bandura [94] has previously highlighted that self-efficacy influences behavior and performance

through various means, with job satisfaction being a significant pathway. Wang et al. [115] also underscore the importance of job satisfaction in enhancing work engagement among teachers.

When teachers feel confident in their work, which is indicative of high self-efficacy, they are more likely to experience satisfaction in their professional roles and work environment. This satisfaction, in turn, enhances work engagement during their teaching activities. Thus, job satisfaction serves as a direct manifestation of teachers' self-efficacy. Confident teachers derive recognition and fulfillment from their efforts and abilities, reinforcing their self-efficacy and ultimately increasing their work engagement. Therefore, hypothesis 2 proposed in this study has been substantiated, highlighting job satisfaction's crucial role in influencing work engagement.

6. Conclusion

Based on the findings of this study, self-efficacy emerged as a significant indirect predictor of work engagement among teachers in economically disadvantaged areas, with job satisfaction playing a crucial mediating role. The results support that enhancing self-efficacy can effectively increase job satisfaction, thereby improving overall work engagement. Thus, fostering both self-efficacy and job satisfaction is crucial for promoting work engagement among teachers in economically disadvantaged settings.

7. Limitations and suggestions for future research

However, this study also had some limitations.

Firstly, the participants used in this study were only distributed in a single area (Hubei Province, China) and cannot represent all the teachers in economically disadvantaged areas worldwide. Therefore, generalizing the results to all teachers in economically disadvantaged areas globally would be difficult.

Secondly, the method used in this study was solely a quantitative questionnaire survey. A qualitative survey of teachers' opinions would have been beneficial to formulate targeted strategies.

Thirdly, this investigation employed a cross-sectional design, analyzing the relationships among variables with one-time collected data, which limits the analysis of causation between variables. Although structural equation model analysis can partially provide insight into understanding the relationships between variables, it cannot reveal causation.

Fourthly, this investigation only focused on the relationships between self-efficacy, job satisfaction, and work engagement for teachers in economically disadvantaged areas, without covering other factors that may affect these variables, such as school management, colleague support, and family background. These unconsidered factors may play roles in explaining the self-efficacy, job satisfaction, and work engagement of teachers.

To the best of our knowledge, this was the first study to explore the role of job satisfaction and self-efficacy in improving work engagement in economically disadvantaged areas. This study provided evidence for implementing strategies to increase work engagement among teachers in economically disadvantaged areas, aiming to optimize educational outcomes and reduce educational resource disparities between developed and underdeveloped areas. Future surveys should conduct in-depth interviews and focus group surveys with teachers in economically disadvantaged areas, which would help to better understand their thoughts. Further research on other parameters, such as school management, colleague support, and family background, are needed. Therefore, our study should be considered exploratory.

8. Contribution of the research

The findings of this research underscore the importance of recognizing teachers in economically disadvantaged areas as key stakeholders whose self-efficacy and job satisfaction significantly impact work engagement. Educational administrators and policymakers should prioritize initiatives that enhance teachers' self-efficacy, as it empowers them to effectively navigate challenges and maintain job satisfaction. Additionally, fostering a supportive work environment that promotes job satisfaction is crucial for sustaining

teachers' engagement and commitment to their educational roles. By investing in strategies that bolster self-efficacy and job satisfaction among teachers, educational institutions can foster a more engaged and effective teaching workforce, ultimately enhancing educational outcomes in economically disadvantaged areas.

9. Conflict of Interest

All the authors declare that they have no conflict of interest.

10. Declaration of using Generative Artificial Intelligence (AI) and AI-assisted technologies

The authors declare they have not used any of the Generative AI tools in creating this article.

11. Data Availability Statement

The data that support the findings of this study are available from the corresponding author, Shi Qi Xu (xsq941214@naver.com), upon reasonable request.

12. Author Contributions Statement

Hongping Zhou: original draft, conceptualization, methodology, validation, formal analysis, data curation, writing - review & editing, supervision, project administration; De Xin Dang: project administration, methodology, writing - original draft, conceptualization; Shi Qi Xu: writing - original draft, writing - review & editing, supervision, project administration, methodology; Dong-Hwa Chung: project administration, methodology, methodology, formal analysis

13. Funding

This study was supported by a grant from the National Social Science Fund of China (Research on the Occupational Ecological Dilemma and Collaborative Governance of Rural Physical Education Teachers in the New Era; agreement number 21BTY101).

14. References

1. Gao Y, He Q, Liu Y, Zhang L, Wang H, Cai, E. Imbalance in spatial accessibility to primary and secondary schools in china: Guidance for education sustainability. Sustainability. 2016;8: 1236.
2. Mongkolhutthi P. Inequality and imbalance of professional development opportunities: The case of a higher educational institution in Southeast Asia. Journal of Applied Research in Higher Education. 2019;11: 452-465.
3. Biddle C, Azano, AP. Constructing and reconstructing the "rural school problem" a century of rural education research. Review of Research in Education. 2016;40: 298-325.
4. Gordon D, Blundell C, Mills R, Bourke T. Teacher self-efficacy and reform: a systematic literature review. The Australian Educational Researcher. 2023;50: 801-821.
5. Ainley J, Carstens R. Teaching and learning international survey (TALIS) 2018 conceptual framework. 2018;11: 12. Available from: https://www.oecd-ilibrary.org/docserver/799337c2-en.pdf?expires=1721741882&id=id&accname=guest&checksum=152DFE5CF81DA7A0006DAD378E01F111
6. Perera HN, Granziera H, McIlveen P. Profiles of teacher personality and relations with teacher self-efficacy, work engagement, and job satisfaction. Personality and individual Differences. 2018;120: 171-178.
7. Xiong Y, Sun XY, Liu XQ, Wang P, Zheng B. The influence of self-efficacy and work input on physical education teachers' creative teaching. Frontiers in Psychology. 2020;10: 2856.
8. Cai Y, Wang L, Bi Y, Tang R. How can the professional community influence teachers' work engagement? The mediating role of teacher self-efficacy. Sustainability. 2022;14: 10029.
9. Liu E, Huang J. Occupational self-efficacy, organizational commitment, and work engagement. Social Behavior and Personality: an international journal. 2019;47: 1-7.
10. Lemon N, Garvis S. Pre-service teacher self-efficacy in digital technology. Teachers and Teaching.

2016;22: 387-408.

11. Kent AM, Giles RM. Preservice Teachers' Technology Self-Efficacy. SRATE Journal. 2017;26: 9-20.

12. Wagner T, Imanel-Noy D. Are they genuinely novice teachers?: Motivations and self-efficacy of those who choose teaching as a second career. Australian Journal of Teacher Education (Online). 2014;39: 31-57.

13. Demır, S. The role of self-efficacy in job satisfaction, organizational commitment, motivation and job involvement. Eurasian Journal of Educational Research. 2020;20: 205-224.

14. Burić I, Macuka I. Self-efficacy, emotions and work engagement among teachers: A two wave cross-lagged analysis. Journal of Happiness Studies. 2018;19: 1917-1933.

15. Garg K, Dar IA, Mishra M. Job satisfaction and work engagement: A study using private sector bank managers. Advances in Developing Human Resources. 2018;20: 58-71.

16. Li M, Wang Z, Gao J, You X. Proactive personality and job satisfaction: The mediating effects of self-efficacy and work engagement in teachers. Current Psychology. 2017;36: 48-55.

17. D'Amico A, Geraci A, Tarantino C. The relationship between perceived emotional intelligence, work engagement, job satisfaction, and burnout in Italian school teachers: an exploratory study. Psihologijske teme. 2020;29: 63-84.

18. Park KA, Johnson KR. Job Satisfaction, Work Engagement, and Turnover Intention of CTE Health Science Teachers. International journal for research in vocational education and training. 2019;6: 224-242.

19. Oubibi M, Fute A, Xiao W, Sun B, Zhou Y. Perceived organizational support and career satisfaction among Chinese teachers: the mediation effects of job crafting and work engagement during COVID-19. Sustainability. 2022;14: 623.

20. Fute A, Oubibi M, Sun B, Zhou Y, Xiao W. Work values predict job satisfaction among Chinese teachers during COVID-19: the mediation role of work engagement. Sustainability. 2022;14: 1353.

21. Bakker AB, Bal MP. Weekly work engagement and performance: A study among starting teachers. Journal of occupational and organizational psychology. 2010;83: 189-206.

22. Shu K. Teachers' commitment and self-efficacy as predictors of work engagement and well-being. Frontiers in Psychology. 2022;13: 850204.

23. Wang L. Exploring the relationship among teacher emotional intelligence, work engagement, teacher self-efficacy, and student academic achievement: A moderated mediation model. Frontiers in psychology. 2022;12: 810559.

24. Zhen X, Liu Q. The impact of a teacher's professional identity on his/her occupational happiness: The mediating role of psychological capital. Nurture. 2023;17: 394-406.

25. Caprara GV, Barbaranelli C, Borgogni L, Petitta L, Rubinacci A. Teachers', school staff's and parents' efficacy beliefs as determinants of attitudes toward school. European journal of psychology of education. 2003;18: 15-31.

26. Judge TA, Thoresen CJ, Bono JE, Patton GK. The job satisfaction–job performance relationship: A qualitative and quantitative review. Psychological bulletin. 2001;127: 376.

27. Skaalvik EM, Skaalvik S. Teacher job satisfaction and motivation to leave the teaching profession: Relations with school context, feeling of belonging, and emotional exhaustion. Teaching and teacher education. 2011;27: 1029-1038.

28. Weiqi C. The structure of secondary school teacher job satisfaction and its relationship with attrition and work enthusiasm. Chinese Education & Society. 2007;40: 17-31.

29. Bandura A. Self-efficacy mechanism in human agency. American psychologist. 1982;37: 122.

30, Tschannen-Moran M, Hoy AW, Hoy WK. Teacher efficacy: Its meaning and measure. Review of educational research. 1998;68: 202-248.

31. Skaalvik EM, Skaalvik S. Teacher self-efficacy and perceived autonomy: Relations with teacher engagement, job satisfaction, and emotional exhaustion. Psychological reports. 2014;114: 68-77.

32. Klassen RM, Usher EL, Bong M. Teachers' collective efficacy, job satisfaction, and job stress in cross-cultural context. The Journal of Experimental Education. 2010;78: 464-486.

33. Hoang T, Wyatt M. Exploring the self-efficacy beliefs of Vietnamese pre-service teachers of English as a foreign language. System. 2021;96: 102422.

34. Fathi J, Derakhshan A. Teacher self-efficacy and emotional regulation as predictors of teaching stress: An investigation of Iranian English language teachers. Teaching English Language. 2019;13: 117-143.

35. Bentea CC. Teacher self-efficacy, teacher burnout and psychological well-being. European Proceedings of Social and Behavioural Sciences. In: Soare E, Langa C, editors. Education Facing Contemporary World Issues. Future Academy; 2017. pp. 1128-1136.

36. Goddard Y, Kim M. Examining connections between teacher perceptions of collaboration, differentiated instruction, and teacher efficacy. Teachers College Record. 2018;120: 1-24.

37. Skaalvik EM, Skaalvik S. Teacher stress and teacher self-efficacy: Relations and consequences. In T. M. Mclntyre, S. E. Mclntyre, & D. J. Francis (Eds.), Educator Stress: An Occupational Health Perspective. 2017;101-125.

38. Shahzad K, Naureen S. Impact of Teacher Self-Efficacy on Secondary School Students' Academic Achievement. Journal of Education and Educational Development. 2017;4: 48-72.

39. Tschannen-Moran M, Hoy AW. Teacher efficacy: Capturing an elusive construct. Teaching and teacher education. 2001;17: 783-805.

40. Burroughs N, Gardner J, Lee Y, Guo S, Touitou I, Jansen K, Schmidt W. A review of the literature on teacher effectiveness and student outcomes. In: Burroughs N, Gardner J, Lee Y, Guo S, Touitou I, Jansen K, Schmidt W, editors. Teaching for excellence and equity: Analyzing teacher characteristics, behaviors and student outcomes with TIMSS. Springer Cham; 2019. pp. 7-17.

41. Magsucang G, Bacuno SM, Canon R, Piamonte E, Tintin J, Barza J. Effectiveness of Teacher’s Teaching Strategies on Academic Performance of Selected Grade 12 General Academic Strand Students in Bestlink College of the Philippines. Ascendens Asia Singapore–Bestlink College of the Philippines Journal of Multidisciplinary Research. 2020;2: 1.

42. Tomaszewski W, Xiang N, Huang Y, Western M, McCourt B, McCarthy I. The impact of effective teaching practices on academic achievement when mediated by student engagement: Evidence from Australian high schools. Education Sciences. 2022;12: 358.

43. Brophy J. Classroom management techniques. Education and urban society. 1986;18: 182-194.

44. Mansor AN, Eng WK, Rasul MS, Hamzah MIM, Hamid AHA. Effective classroom management. International Education Studies. 2012;5: 35-42.

45. Gage NA, ScottT, Hirn R, MacSuga-Gage AS. The relationship between teachers' implementation of classroom management practices and student behavior in elementary school. Behavioral disorders. 2018;43: 302-315.

46. Aloe AM, Amo LC, Shanahan ME. Classroom management self-efficacy and burnout: A multivariate meta-analysis. Educational psychology review. 2014;26: 101-126.

47. Celis JA, Tan DA, Celis Jr VG, Tan RJ. Classroom Management, Job Satisfaction, and Stress Coping Mechanism on the Performance of Senior High School Teachers. American Journal of Educational Research. 2023;11: 395-406.

48. Sarıtepeci M, Çakır H. The effect of blended learning environments on student motivation and student engagement: a study on social studies course. Education & Science/Egitim ve Bilim, 2015;40: 177.

49. Spector PE. Job satisfaction: Application, assessment, causes, and consequences. Sage; 1997.

50. Weiss HM. Deconstructing job satisfaction: Separating evaluations, beliefs and affective experiences. Human resource management review. 2002;12: 173-194.

51. Zembylas M, Papanastasiou E. Job satisfaction among school teachers in Cyprus. Journal of educational administration. 2004;42: 357-374.

52. Skaalvik EM, Skaalvik S. Teacher self-efficacy and teacher burnout: A study of relations. Teaching and teacher education. 2010;26: 1059-1069.

53. Madigan DJ, Kim LE. Does teacher burnout affect students? A systematic review of its association with

academic achievement and student-reported outcomes. International journal of educational research. 2021;105: 101714.

54. Malik ME, Nawab S, Naeem B, Danish RQ. Job satisfaction and organizational commitment of university teachers in public sector of Pakistan. International journal of business and management. 2010;5: 17.

55. Klassen RM, Chiu MM. Effects on teachers' self-efficacy and job satisfaction: Teacher gender, years of experience, and job stress. Journal of educational Psychology. 2010;102: 741.

56. Ferguson K, Frost L, Hall D. Predicting teacher anxiety, depression, and job satisfaction. Journal of teaching and learning. 2012;8: 1.

57. Tabancalı E. The relationship between teachers' job satisfaction and loneliness at the workplace. Eurasian Journal of Educational Research. 2016;16: 263-280.

58. Edinger SK, Edinger MJ. Improving teacher job satisfaction: The roles of social capital, teacher efficacy, and support. The Journal of Psychology. 2018;152: 573-593.

59. Dinham S, Scott C. A three domain model of teacher and school executive career satisfaction. Journal of educational administration. 1998;36: 362-378.

60. Scott-Ladd BD. The influence of participation in decision-making within the enterprise bargaining context: Implications for job satisfaction and affective commitment. PhD. Thesis, Curtin University. 2001. Available from: https://espace.curtin.edu.au/handle/20.500.11937/2145

61. Huang PY, Wang CC, Tseng YY, Wang RJ. The impact of curriculum design on learning satisfaction. Journal of Information and Optimization Sciences. 2011;32: 637-655.

62. Iqbal S, Guohao L, Akhtar S. Effects of job organizational culture, benefits, salary on job satisfaction ultimately affecting employee retention. Review of Public Administration and Management. 2017;5: 1-7.

63. Fadeyi VT, Sofoluwe AO, Gbadeyan RA. Influence of teachers' welfare scheme on job performance in selected Kwara State Secondary Schools. Asia Pacific Journal of Education, Arts and Sciences. 2015;2: 89-93.

64. Naluwemba F, Sekiwu D, Vincent O. The interplay of school welfare provision and teacher performance: The case of Ugandan secondary schools, International Journal of Educational Policy Research and Review. 2016;3: 6-13.

65. Sims S. Modelling the relationships between teacher working conditions, job satisfaction and workplace mobility. British Educational Research Journal. 2020;46: 301-320.

66. Ostad SA, Ghanizadeh A, Ghanizadeh M. The dynamism of EFL teachers' professional identity with respect to their teaching commitment and job satisfaction. Cogent Education. 2019;6: 1685353.

67. Huang S, Huang Y, Chang W, Chang L, Kao P. Exploring the effects of teacher job satisfaction on teaching effectiveness. In International Journal of Modern Education Forum. 2013;2: 17-30.

68. Bogler R. The influence of leadership style on teacher job satisfaction. Educational administration quarterly. 2001;37: 662-683.

69. Khaliq A. Effect of salary, promotion, and relationships with colleagues on secondary school teachers' job satisfaction. Pakistan Journal of Educational Research and Evaluation (PJERE). 2021;4: 1-20.

70. Shahzad MN, Ali W, Shafiq A. Relationship of Teachers' Social Recognition with their Self-Esteem at the Secondary School Level. Voyage Journal of Educational Studies. 2024;4: 28-41.

71. Bakker AB, Demerouti E, Verbeke W. Using the job demands-resources model to predict burnout and performance. Human Resource Management: Published in Cooperation with the School of Business Administration, The University of Michigan and in alliance with the Society of Human Resources Management. 2004;43: 83-104.

72. Trépanier SG, Fernet C, Austin S, Ménard J. Revisiting the interplay between burnout and work engagement: An exploratory structural equation modeling (ESEM) approach. Burnout Research. 2015;2: 51-59.

73. Jia K, Zhu T, Zhang W, Rasool SF, Asghar A, Chin T. The linkage between ethical leadership, well-being, work engagement, and innovative work behavior: the empirical evidence from the higher education sector

of China. International journal of environmental research and public health. 2022;19: 5414.

74. Schaufeli WB, Salanova M, González-Romá V, Bakker AB. The measurement of engagement and burnout: A two sample confirmatory factor analytic approach. Journal of Happiness studies. 2002;3: 71-92.

75. Bakker AB, Demerouti E, Sanz-Vergel AI. Burnout and work engagement: The JD–R approach. Annu. Rev. Organ. Psychol. Organ. Behav. 2014;1: 389-411.

76. Bakker AB, Albrecht SL, Leiter MP. Key questions regarding work engagement. European journal of work and organizational psychology. 2011;20: 4-28.

77. Tisu L, Vîrgă D, Mermeze I. Autonomy and performance: Proactive vitality management and work engagement as sequential mediators of the relationship. Psychological reports. 2023;126: 411-433.

78. Guglielmi D, Avanzi L, Chiesa R, Mariani MG, Bruni I, Depolo M. Positive aging in demanding workplaces: The gain cycle between job satisfaction and work engagement. Frontiers in psychology. 2016;7: 1224.

79. Radic A, Arjona-Fuentes JM, Ariza-Montes A, Han H, Law R. Job demands–job resources (JD-R) model, work engagement, and well-being of cruise ship employees. International Journal of Hospitality Management. 2020;88: 102518.

80. Sujono D, Tunas B, Sudiarditha I. Vitality of work involvement in mediation: The effect of organizational justice on organizational citizenship behavior. Management Science Letters. 2020;10: 1061-1068.

81. Schaufeli WB, Bakker AB. Defining and measuring work engagement: Bringing clarity to the concept. Work engagement: A handbook of essential theory and research, 12, 10-24.82. Macey, W. H., & Schneider, B. (2008). The meaning of employee engagement. Industrial and organizational Psychology. 2010;1: 3-30.

83. Saks AM. Antecedents and consequences of employee engagement. Journal of managerial psychology. 2006;21: 600-619.

84. Tims M, Bakker AB, Derks D, Van Rhenen W. Job crafting at the team and individual level: Implications for work engagement and performance. Group & Organization Management. 2013;38: 427-454.

85. Kašpárková L, Vaculík M, Procházka J, Schaufeli WB. Why resilient workers perform better: The roles of job satisfaction and work engagement. Journal of Workplace Behavioral Health. 2018;33: 43-62.

86. Arifin Z, Nirwanto N, Manan A. Improving the effect of work satisfaction on job performance through employee engagement. International Journal of Multi-Discipline Science (IJ-MDS). 2019;2: 1-9.

87. Tims M, Bakker AB, Xanthopoulou D. Do transformational leaders enhance their followers' daily work engagement?. The leadership quarterly 2011;22: 121-131.

88. Zakariya YF. Effects of school climate and teacher self-efficacy on job satisfaction of mostly STEM teachers: A structural multigroup invariance approach. International Journal of STEM Education. 2020;7: 1-12.

89. Yoon I, Kim M. Dynamic patterns of teachers' professional development participation and their relations with socio-demographic characteristics, teacher self-efficacy, and job satisfaction. Teaching and Teacher Education. 2022;109: 103565.

90. Burić I, Kim LE. Job satisfaction predicts teacher self-efficacy and the association is invariant: Examinations using TALIS 2018 data and longitudinal Croatian data. Teaching and Teacher Education. 2021;105: 103406.

91. Kalkan F. The Relationship Between Teachers' Self-Efficacy Beliefs and Job Satisfaction Levels: A Meta-Analysis Study. Education & Science/Egitim ve Bilim. 2020;45: 219787701.

92. Ismayilova K, Klassen RM. Research and teaching self-efficacy of university faculty: Relations with job satisfaction. International Journal of Educational Research. 2019;98: 55-66.

93. Egyed CJ, Short RJ. Teacher self-efficacy, burnout, experience and decision to refer a disruptive student. School psychology international. 2006;27: 462-474.

94. Bandura A. Self-efficacy: The exercise of control. Worth Publishers; 1997.

95. Klassen RM, Aldhafri S, Mansfield CF, Purwanto E, Siu AF, Wong MW, Woods-McConney A. Teachers' engagement at work: An international validation study. The journal of experimental education. 2012;80:

317-337.
96. Eldor L, Shoshani A. Caring relationships in school staff: Exploring the link between compassion and teacher work engagement. Teaching and Teacher Education. 2016;59: 126-136.
97. Boštjančič E, Petrovčič A. Exploring the relationship between job satisfaction, work engagement and career satisfaction: The study from public university. Human systems management. 2019;38: 411-422.
98. Skaalvik EM, Skaalvik S. Teachers' perceptions of the school goal structure: Relations with teachers' goal orientations, work engagement, and job satisfaction. International Journal of Educational Research. 2013;62: 199-209.
99. Høigaard R, Giske R, Sundsli K. Newly qualified teachers' work engagement and teacher efficacy influences on job satisfaction, burnout, and the intention to quit. European Journal of Teacher Education. 2012;35: 347-357.
100. Timms C, Brough P. "I like being a teacher" Career satisfaction, the work environment and work engagement. Journal of Educational Administration. 2013;51: 768-789.
101. De Simone S, Planta A, Cicotto G. The role of job satisfaction, work engagement, self-efficacy and agentic capacities on nurses' turnover intention and patient satisfaction. Applied Nursing Research. 2018;39: 130-140.
102. Yakın M, Erdil O. Relationships between self-efficacy and work engagement and the effects on job satisfaction: a survey on certified public accountants. Procedia-Social and Behavioral Sciences. 2012;58: 370-378.
103. Chen S, Sun S. A study on the job satisfaction and influencing factors of ideological and political teachers in colleges and universities. Journal of Guangxi Education Institute. 2023;4: 114-121.
104. Bian J. The relationship between social support, professional identity and job satisfaction of primary and secondary school physical education teachers - based on sociological empirical research. Abstracts of the 13th National Sports Science Conference - Special Report. 2023;627-629.

105. Huang Z. A study on job satisfaction of full-time teachers in private universities based on multiple regression analysis: Taking MD College as an example. Market Weekly. 2024;1: 179-182.

106. Umar HIS, Usman M. The imperative of population sampling in social science research. Global Journal of Political and Science and Administration. 2015;3: 49-57.

107. Xu SQ, Zhou L, Kim SH, Chung DH, Li Z. Internal marketing analysis for improving the internal consumer satisfaction and customer orientation of employees in private-owned sports center. Plos one. 2023;18: e0286021.

108. Chahal H, Mehta S. Modeling patient satisfaction construct in the Indian health care context. International Journal of Pharmaceutical and Healthcare Marketing. 2013;7: 75-92.

109. Anderson JC, Gerbing DW. Structural equation modeling in practice: A review and recommended two-step approach. Psychological bulletin. 1988;103: 411.

110. Bajaj B, Pande N. Mediating role of resilience in the impact of mindfulness on life satisfaction and affect as indices of subjective well-being. Personality and individual differences. 2016;93: 63-67.

111. Ortan F, Simut C, Simut R. Self-efficacy, job satisfaction and teacher well-being in the K-12 educational system. International journal of environmental research and public health. 2021;18: 12763.

112. Türkoglu ME, Cansoy R, Parlar H. Examining Relationship between Teachers' Self-Efficacy and Job Satisfaction. Universal journal of educational research. 2017;5: 765-772.

113. Oubibi M, Fute A, Xiao W, Sun B, Zhou Y. Perceived organizational support and career satisfaction among Chinese teachers: the mediation effects of job crafting and work engagement during COVID-19. Sustainability. 2022;14: 623.

114. Hakanen JJ, Bakker AB, Schaufeli WB. Burnout and work engagement among teachers. Journal of school psychology. 2006;43: 495-513.

115. Wang Z, Jiang H, Jin W, Jiang J, Liu J, Guan J, Liu Y, Bin E. Examining the antecedents of novice stem teachers' job satisfaction: The roles of personality traits, perceived social support, and work engagement.

Behavioral Sciences. 2024;14: 214.

Click here to access/download
Supporting Information
Supplementary Fig 3.docx

Click here to access/download
Supporting Information
Supplementary Fig 2.docx

Click here to access/download
Supporting Information
Supplementary Fig 1.docx

Approval letter

Click here to access/download
Supporting Information
renamed_4f4ed.pdf

Click here to access/download
Supporting Information
Approval letter translation.docx
