# Supplementary material for: Job satisfaction mediates the effect of self-efficacy on work engagement among physical education teachers in economically disadvantaged areas
Source: PLoS One. 2025 Apr 17;20(4):e0321055. doi: 10.1371/journal.pone.0321055 (PMC12005525; doi:10.1371/journal.pone.0321055)
Supplement: Attachment — Submitted filename: Response to Reviewers.docx [file pone.0321055.s007.docx]

Dear editor and honored reviewers

We really appreciate you and the reviewers for your comments on our Manuscript: “**Job satisfaction mediates the effect of self-efficacy on work engagement among physical education teachers in economically disadvantaged areas”** We express our sincere gratitude and thankfulness for your time and precision in reviewing our manuscript. The responses to the comments are as follows. For your kind information, we have carefully dealt with the comments of the reviewers as follows. We hope the revised manuscript meets the standard of publication. Thank you.

A.Title and Scope Clarification

The title should be revised to reflect that the study is focused on physical education teachers in a specific context. The introduction, literature review, abstract, discussion, and conclusions should consistently emphasize that the findings are limited to physical education teachers and the specific sample used in the study. This will help avoid contradictions regarding the generalizability of the study.

**Response:** Thank you for your valuable comments. Based on the editor’s and reviewers’ feedback, we have revised the title to: Job satisfaction mediates the effect of self-efficacy on work engagement among physical education teachers in economically disadvantaged areas.

Moreover, we have emphasized that this study is limited to physical education teachers in economically disadvantaged areas in the introduction, literature review, abstract, and other relevant sections. The overall manuscript has been revised to eliminate contradictions regarding the study’s generalizability, as you mentioned. Thanks so much.

B.Sample and Methodology

•	Sample Size Calculation: The method used for calculating the sample size should be clearly explained to enhance transparency and methodological rigor.

**Response:** Thank you for your valuable comments. We have declared the method used for calculating the sample size.

“Before participation, all respondents were informed about the survey’s purpose and provided written informed consent. Data collection occurred from 21 March 2022 to 28 May 2023. The sample size was determined using PASS software (version 15.0.5) with a two-sided confidence interval method. A confidence interval width of 0.1, a confidence level of 0.95, an acceptance rate of 50%, and a dropout rate of 10% were used, indicating that at least 428 valid questionnaires were needed. The 472 valid responses exceeded the required sample size [104,105].”

Please refer to Line 224-229. Thanks so much.

•	Study Scope: There is concern about the limited scope of the study, which focuses solely on physical education teachers, while the manuscript suggests a broader scope. The authors should clarify this limitation throughout the manuscript.

**Response:** Thank you for your valuable comments. We have emphasized throughout the manuscript that this study is limited to physical education teachers in economically disadvantaged areas. Additionally, we have acknowledged this limitation. Please refer to Line 329-332.

“First, the sample was limited to PE teachers in economically disadvantaged areas of Hubei Province, China, which restricts the generalizability of the findings to PE teachers in economically disadvantaged areas worldwide. Future research should include a more diverse and geographically representative sample to enhance external validity.”

Please check it. Thanks so much.

•	Inclusion and Exclusion Criteria: The inclusion and exclusion criteria for the study should be stated clearly in the methodology section to ensure proper understanding of the study's sample.

**Response:** Thank you for your valuable comments. We have disclosed the inclusion and exclusion criteria in Lines 220–222.

“Inclusion criteria required participants to have at least one year of teaching experience and be willing to participate in the survey. Exclusion criteria included dissatisfaction with participation, non-teaching staff, administrators, and incomplete or unreliable responses.”

Please check it on the Line 220-222. Thanks so much.

C.Data and Results Presentation

•	Presentation of Results: The results section should avoid unscientific language (e.g., "investigation" and "educationally") and ensure that terms are precise and adhere to scientific standards.

**Response:** Thank you for your valuable comments. We had two native speakers review and revise the manuscript for grammar. The mistakes you mentioned have been corrected. We hope the grammar now meets your expectations. Thanks so much.

•	Questionnaire and Data Analysis: The questionnaire could be placed in an appendix rather than as a table within the main text. The validity and reliability of the questionnaire need to be addressed, particularly if it is to be applied in other contexts. A detailed explanation of the factors considered in the analysis should be provided.

**Response:** Thank you for your valuable comments. We agree with your feedback. The questionnaire has been submitted as a supplementary file. Additionally, the validity and reliability analysis of the questionnaire is provided in Lines 260–266.

“Factor analysis of self-efficacy (S1 Fig), job satisfaction (S2 Fig), and work engagement (S3 Fig) revealed no questions with low factor loading values, confirming the adequacy of the measurement items. The Cronbach’s α coefficients for all factors supported the internal consistency of the scales. Additionally, the convergent and discriminant validity statistics showed that the average variance extracted and composite reliability values for all multi-item scales exceeded the thresholds of 0.5 and 0.8, respectively, indicating satisfactory convergent validity for the measurement model.”

Please check it. Thanks so much.

For a detailed explanation of the factors considered in the analysis, please see the Statistical Analysis section in Lines 232–235.

“The validity and reliability of the questionnaire were assessed through confirmatory factor analysis and Cronbach’s α [106]. Convergent validity was deemed satisfactory for multi-item scales if the average variance extracted value exceeded 0.5 and composite reliability exceeded 0.8 [107].”

Thanks so much.

•	Standardization of Data: There are discrepancies in the data presented, such as inconsistencies in table formatting and numerical values. The authors should standardize the data and correct errors in presentation.

**Response:** Thank you for your valuable comments. We sincerely apologize for the data-related mistake. The manuscript has been thoroughly checked, and the relevant errors have been corrected. Thanks so much.

D.Introduction and Literature Review

•	Tighten Introduction: The introduction is overly long and should be shortened to improve clarity and focus. It should directly address the research gap and the study's objectives.

**Response:** Thank you for your valuable comments. We have restructured the Introduction section to enhance clarity and focus. Please refer to Lines 32–48. Thanks so much.

•	Literature Review: Redundant content between the introduction and literature review should be removed to improve the flow and coherence of the manuscript. The literature review should also integrate findings relevant to the specific context of physical education.

**Response:** Thank you for your valuable comments. We have removed redundant content between the Introduction and Literature Review sections, as you suggested. Furthermore, we have incorporated relevant findings related to physical education into the literature review. Please check it. Thanks so much.

E.Formatting and Structural Issues

•	Formatting: The manuscript should adhere to PLOS ONE guidelines, particularly regarding formatting issues such as authors' affiliations on the title page, indentation in paragraphs, and titles. Proper attention to these details is necessary to ensure the manuscript meets submission standards.

**Response:** Thank you for your valuable comments. We have reformatted the manuscript to comply with the style guidelines of PLOS ONE. We apologize for the previous formatting errors.

•	Abbreviations: Ensure that all abbreviations are fully spelled out the first time they are used in the manuscript.

**Response:** Thank you for your valuable comments. We have ensured that all abbreviations are fully spelled out upon first mention in the manuscript. Thanks so much.

•	Figures and Tables: All figures should meet the PLOS ONE guidelines. It is suggested that Tables 6-11 be consolidated into a single table for better clarity. Figures and tables should also be clearly labeled and referenced within the text.

**Response:** Thank you for your valuable comments. The citation style has been revised to adhere to the PLOS ONE format.

Additionally, we do not have Tables 6–11. All figures and tables are now clearly labeled and properly referenced in the manuscript

Please check it. Thanks so much.

F.Discussion and Conclusion

•	Discussion of Results: The discussion section should not merely repeat results but should interpret the findings in light of existing literature. It should highlight the implications of the findings, including any possible generalizations, and discuss the significance of the results.

**Response:** Thank you for your valuable comments. We have reorganized the Discussion section. Please check this part. Thanks so much.

•	Conclusions: The conclusions should be grounded in the study's findings and should avoid overstatements. Recommendations should be specific and related to the study context, such as suggestions for improving laboratory conditions or teaching practices in physical education.

**Response:** Thank you for your valuable comments. We have revised the Conclusion section. The suggested contributions have been integrated into the Research Contribution section. Please check it. Thanks so much.

G.Typographical and Grammatical Corrections

The manuscript contains numerous typographical, grammatical, and small formatting errors. The authors are encouraged to thoroughly revise the manuscript to ensure proper language use and consistency throughout the document.

**Response:** Thank you for your valuable comments. We had two native speakers review the grammar of this manuscript. Additionally, we reformatted the manuscript according to PLOS ONE guidelines. Please check it. Thanks so much.

Journal Requirements:

1. When submitting your revision, we need you to address these additional requirements.

Please ensure that your manuscript meets PLOS ONE's style requirements, including those for file naming. The PLOS ONE style templates can be found at

https://journals.plos.org/plosone/s/file?id=wjVg/PLOSOne_formatting_sample_main_body.pdf and

https://journals.plos.org/plosone/s/file?id=ba62/PLOSOne_formatting_sample_title_authors_affiliations.pdf

**Response:** Thank you for your valuable comments. We have reformatted the manuscript to comply with the style guidelines of PLOS ONE. We apologize for the previous formatting errors.

2. Thank you for stating the following financial disclosure:

[This study was supported by a grant from the National Social Science Fund of China (Research on the Occupational Ecological Dilemma and Collaborative Governance of Rural Physical Education Teachers in the New Era; agreement number 21BTY101)].

Please state what role the funders took in the study. If the funders had no role, please state: "The funders had no role in study design, data collection and analysis, decision to publish, or preparation of the manuscript."

If this statement is not correct you must amend it as needed.

Please include this amended Role of Funder statement in your cover letter; we will change the online submission form on your behalf.

**Response:** Thank you for your valuable comments. This statement was incorrect. The correct statement is:

“Hongping Zhou: original draft, conceptualization, methodology, validation, formal analysis, data curation, writing - review & editing, supervision, project administration; De Xin Dang: project administration, methodology, writing - original draft, conceptualization; Shi Qi Xu: writing - original draft, writing - review & editing, supervision, project administration, methodology; Dong-Hwa Chung: project administration, methodology, formal analysis”

We have disclosed this information in the Author Contributions section. Please check it. Thanks so much.

3. In this instance it seems there may be acceptable restrictions in place that prevent the public sharing of your minimal data. However, in line with our goal of ensuring long-term data availability to all interested researchers, PLOS’ Data Policy states that authors cannot be the sole named individuals responsible for ensuring data access (http://journals.plos.org/plosone/s/data-availability#loc-acceptable-data-sharing-methods).

Data requests to a non-author institutional point of contact, such as a data access or ethics committee, helps guarantee long term stability and availability of data. Providing interested researchers with a durable point of contact ensures data will be accessible even if an author changes email addresses, institutions, or becomes unavailable to answer requests.

Before we proceed with your manuscript, please also provide non-author contact information (phone/email/hyperlink) for a data access committee, ethics committee, or other institutional body to which data requests may be sent. If no institutional body is available to respond to requests for your minimal data, please consider if there any institutional representatives who did not collaborate in the study, and are not listed as authors on the manuscript, who would be able to hold the data and respond to external requests for data access? If so, please provide their contact information (i.e., email address). Please also provide details on how you will ensure persistent or long-term data storage and availability.

**Response:** Thank you for your valuable comments. We are happy to share our data. All data used for this survey are available at figshare at https://doi.org/10.6084/m9.figshare.28365692.v1. Please check it. Thanks so much.

4. Please include captions for your Supporting Information files at the end of your manuscript, and update any in-text citations to match accordingly. Please see our Supporting Information guidelines for more information: http://journals.plos.org/plosone/s/supporting-information.

**Response:** Thank you for your valuable comments. We have added captions for the supporting information files at the end of the manuscript. Please refer to Line 654-658. Thanks so much.

5. Review Comments to the Author

Reviewer #1: All comments and observations on the work presented by the authors can be found in detail in the attached file. Authors are requested to pay attention to each of the annotations to ensure the quality and clarity of the manuscript.

Line 26: Search for other keywords, 4 of the 5 listed are included in the title.

**Response:** Thank you for your valuable comments. We have revised the keywords to: rural teacher, rural education, teacher motivation, educational policy, burnout prevention

Please refer to Line 30. Thanks so much.

Line 35-36: Add citation.

**Response:** Thank you for your valuable comments. We have added a reference for this sentence. Please refer to Line 37-38. Thanks so much.

“Consequently, enhancing the work engagement of PE teachers in economically disadvantaged areas has become a focal point for many scholars [5].”

Please check it. Thanks again.

Line 48: engagement

**Response:** Thank you for your valuable comments. The relevant mistake has been corrected. Thanks so much.

Line 71-72: Rewrite, it is equal to lines 38 and 39.

**Response:** Thank you for your valuable comments. We have removed these sections based on the editor’s feedback. Thanks so much.

Line 78-79: Rewrite, it is equal to line 40.

**Response:** Thank you for your valuable comments. We have removed these sections based on the editor’s feedback. Thanks so much.

Line 130: “extrinsic” not intrinsic.

**Response:** Thank you for your valuable comments. The relevant mistake has been corrected. Please check the Line 87. Thanks so much.

Line 161-164: The citation does not correspond to what is written in the paragraph.

**Response:** Thank you for your valuable comments. We have corrected this reference. Please check the Line 117-120. Thanks so much.

Fig 1: Instructional_strategies

**Response:** Thank you for your valuable comments. The relevant mistake has been corrected. Please check Fig 1. Thanks so much.

Table 1: Delete this line; Incomplete line; Delete this line; Chen and Sun, 2023

**Response:** Thank you for your valuable comments. The relevant mistake has been corrected. Additionally, Table 1 has been uploaded as a supplementary file. Please check S1 Table. Thanks so much.

Line 262-264: Follow the order indicated in Table 1: course teaching, welfare treatment, work environment, job recognition, and social status.

**Response:** Thank you for your valuable comments. We have revised the order of these parameters. Please check Line 211-213. Thanks so much.

Line 265-266: Mention also reference 81.

**Response:** Thank you for your valuable comments. We have added a reference to this sentence. Please check the Line 214-215. Thanks so much.

Line 266-267: Follow the order indicated in Table 1: vigor, dedication, and absorption.

**Response:** Thank you for your valuable comments. We have revised the order of these parameters. Please check Line 215-216. Thanks so much.

Line 271: Explain in detail.

**Response:** Thank you for your valuable comments. This survey used a convenience sampling method. During the survey period, we visited the above schools and invited the respondents face-to-face.

Relevant details have been provided in Lines 219–220. Please check. Thank you.

Fig 2. Educational background; Contract type; Weekly teaching hours

**Response:** Thank you for your valuable comments. The relevant mistake has been corrected. Please check the Fig 2. Thanks so much.

Line 309: Standardize with line 278.

**Response:** Thank you for your valuable comments. We have standardized the expression of Cronbach’s α in the manuscript. Thanks so much.

Line 310: Standardize with line 279.

**Response:** Thank you for your valuable comments. We have standardized the expression of average variance extracted and composite reliability in the manuscript. Thanks so much.

Line 315-317: Respect the order mentioned in the lines 285-286.

**Response:** Thank you for your valuable comments. We have revised the order of these parameters. Please check Line 268-270. Thanks so much.

Line 339: Remove dot.

**Response:** Thank you for your valuable comments. We have removed this unnecessary dot. Thanks so much.

Line 348: Repeated reference, is the same as number 19, mentioned in the lines 477-479.

**Response:** Thank you for your valuable comments. We sincerely apologize for the mistake. The reference list has been checked and corrected accordingly. Thanks so much.

Line 355: This phrase is repeated several times (four), paraphrase in each case.

**Response:** Thank you for your valuable comments. We have revised this sentence for avoiding repeat. Please check the Line 304. Thanks so much.

Line 431: It is repeated.

**Response:** Thank you for your valuable comments. Repeated content has been removed. Thanks so much.

Line 447-450: Use the citation provided by the official source. Available in: https://doi.org/10.1787/799337c2-en

Line 494-495: Incomplete citation, verify in: https://psycnet.apa.org/doi/10.1037/0033-2909.127.3.376

Line 501: Incomplete citation, verify in: https://psycnet.apa.org/doi/10.1037/0003-066X.37.2.122

Line 546-547: Incomplete citation, verify in: https://egitimvebilim.ted.org.tr/index.php/EB/article/download/2592/966

Line 558-559: Incomplete citation, verify in: https://ccsenet.org/journal/index.php/ijbm/article/download/5204/4901

Line 560-561: Incomplete citation, verify in: https://psycnet.apa.org/doi/10.1037/a0019237

Line 562-563: Incomplete citation, verify in: https://jtl.uwindsor.ca/index.php/jtl/article/view/2896/pdf

Line 570-572: The link to the document is broken and cannot be consulted. Cite the published article of the thesis, available at: https://doi.org/10.1108/01437730410564988

Line 575-576: Incomplete citation, check in: https://www.walshmedicalmedia.com/open-access/effects-of-job-organizational-culture-benefits-salary-on-job-satisfactionultimately-affecting-employee-retention-2315-7844-1000229.pdf

Line 607-608: The name of the journal is written in abbreviated form and not in full, as are the other references.

Line 621-623: Separate references 81 and 82.

Line 634-636: Incomplete citation, check in: https://doi.org/10.1186/s40594-020-00209-4

Line 643-644: Incomplete citation, check in: https://egitimvebilim.ted.org.tr/index.php/EB/article/download/8549/3080

Line 685-686: Incomplete citation, check in: https://psycnet.apa.org/doi/10.1037/0033-2909.103.3.411

Line 693-695: Repeated reference, is the same as number 19, mentioned in the lines 477-479.

**Response:** Thank you for your valuable comments. We have corrected these references based on your feedback. Moreover, we have checked the doi link for all references, and added all the information we can search. Thanks so much.

Reviewer #2: 1. Perhaps the questionnaire should be placed in an appendix rather than a table in the text.

**Response:** Thank you for your valuable comments. The questionnaire has been submitted as a supplementary file. Thanks so much.

2. Why physical education teachers and not other teachers? The introduction and review of the literature are about teachers and not specifically physical education teachers.

**Response:** Thank you for your valuable comments. Teachers in economically disadvantaged areas often exhibit lower levels of work engagement due to constraints in resources and limited opportunities for professional growth. This issue is particularly pronounced among physical education (PE) teachers, as PE is frequently regarded as a marginal subject. As a result, PE teachers receive less recognition from administrators and parents, encounter greater professional challenges, and experience diminished work engagement.

This is why our study focuses on PE teachers. Relevant descriptions have been included in the Abstract and Introduction. Furthermore, we have emphasized throughout the manuscript that this study is limited to PE teachers in economically disadvantaged areas.

Thanks so much.

3. The reference to teachers throughout the text contradicts the restricted sample of physical education teachers used in this study. If any, the findings of this study are limited to physical education teachers only, which you acknowledge in the limitations section.

**Response:** Thank you for your valuable comments. We have emphasized throughout the manuscript that this study is limited to physical education teachers in economically disadvantaged areas. Additionally, we have addressed this limitation. Please check the Line 329-332.

“First, the sample was limited to PE teachers in economically disadvantaged areas of Hubei Province, China, which restricts the generalizability of the findings to PE teachers in economically disadvantaged areas worldwide. Future research should include a more diverse and geographically representative sample to enhance external validity.”

Please check it. Thanks so much.

4. Thus, the title should clearly state “… for physical education teachers in…” and the abstract, introduction, review of the literature, discussion, conclusions, and all relevant sections should reflect this fact.

**Response:** Thank you for your valuable comments. We have revised the title to: Job satisfaction mediates the effect of self-efficacy on work engagement among physical education teachers in economically disadvantaged areas.

Moreover, we have emphasized that this study is limited to physical education teachers in economically disadvantaged areas in the introduction, literature review, abstract, and other relevant sections. The overall manuscript has been revised to eliminate contradictions regarding the study’s generalizability, as you mentioned. Thanks so much.

Reviewer #3: Respected Authors,

The overall quality of your scientific research is good. However, there are some unscientific words like investigation and educationally in the results section.

**Response:** Thank you for your valuable comments. We have invited two native speakers to review and refine the grammar in this manuscript. The mistakes you highlighted have been corrected accordingly. We hope the grammar now meets your expectations. Thanks so much.

Secondly, your introduction is so long that it becomes boring. Please tighten up your introduction section.

**Response:** Thank you for your valuable comments. We have restructured the Introduction to enhance clarity and focus. Additionally, we have removed redundant content between the Introduction and Literature Review, as suggested by you and the editor. Please review the revised Introduction in Lines 32-48. Thanks so much.

Thirdly, please explain the sample size calculation method used for selecting your study sample.

**Response:** Thank you for your valuable comments. We have declared the method used for calculating the sample size.

“Before participation, all respondents were informed about the survey’s purpose and provided written informed consent. Data collection occurred from 21 March 2022 to 28 May 2023. The sample size was determined using PASS software (version 15.0.5) with a two-sided confidence interval method. A confidence interval width of 0.1, a confidence level of 0.95, an acceptance rate of 50%, and a dropout rate of 10% were used, indicating that at least 428 valid questionnaires were needed. The 472 valid responses exceeded the required sample size [104,105].”

Please check the Line 224-229. Thanks so much.

Fourthly, there are some formatting issues with your manuscript, like the authors' affiliations on the title page, indents in the first lines of each and every paragraph, and even some titles. Please review PLOS ONE guidelines to adjust them accordingly.

**Response:** Thank you for your valuable comments. We have reformatted the manuscript to comply with the style guidelines of PLOS ONE. We apologize for the previous formatting errors.

Fifthly, include full descriptions when you are citing the abbreviations for the first time.

**Response:** Thank you for your valuable comments. We have ensured that all abbreviations are fully spelled out upon first mention in the manuscript. Thanks so much.

Sixthly, please ensure all your figures meet the PLOS ONE guidelines.

**Response:** Thank you for your valuable comments. The citation style has been revised to adhere to the PLOS ONE format. All figures and tables are now clearly labeled and properly referenced in the manuscript

Please check it. Thanks so much.
